# Supplementary material for: Agreement between ranking metrics in network meta-analysis: an empirical study
Source: BMJ Open. 2020 Aug 20;10(8):e037744. doi: 10.1136/bmjopen-2020-037744 (PMC7440831; doi:10.1136/bmjopen-2020-037744)
Supplement: Supplementary data [file bmjopen-2020-037744supp001.pdf]

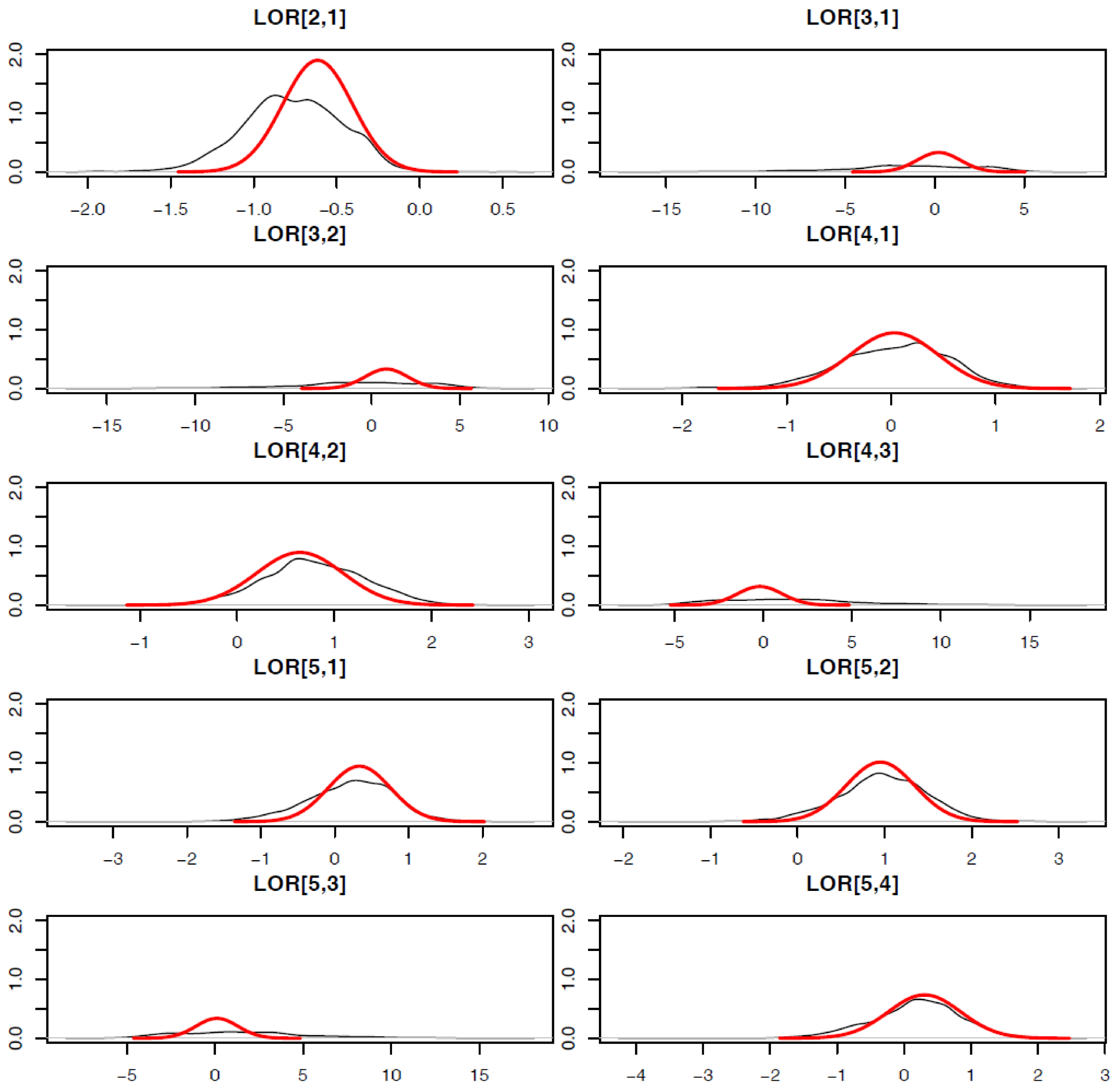

Figure S1: Normal distributions of relative treatment effect estimates from frequentist setting (red line) superimposed on posterior distributions of the relative treatment effects from Bayesian model (black line) for a network with Spearman's ρ of 0.6 between $SUCRA_F$ and $SUCRA_B$. The network meta-analysis used analysed the effects of four inodilators and placebo on survival in adult cardiac surgery patients (Greco et al., *Br J Anaesth* 2015). LOR = log odds ratios; number in square brackets represents the different interventions in the network.

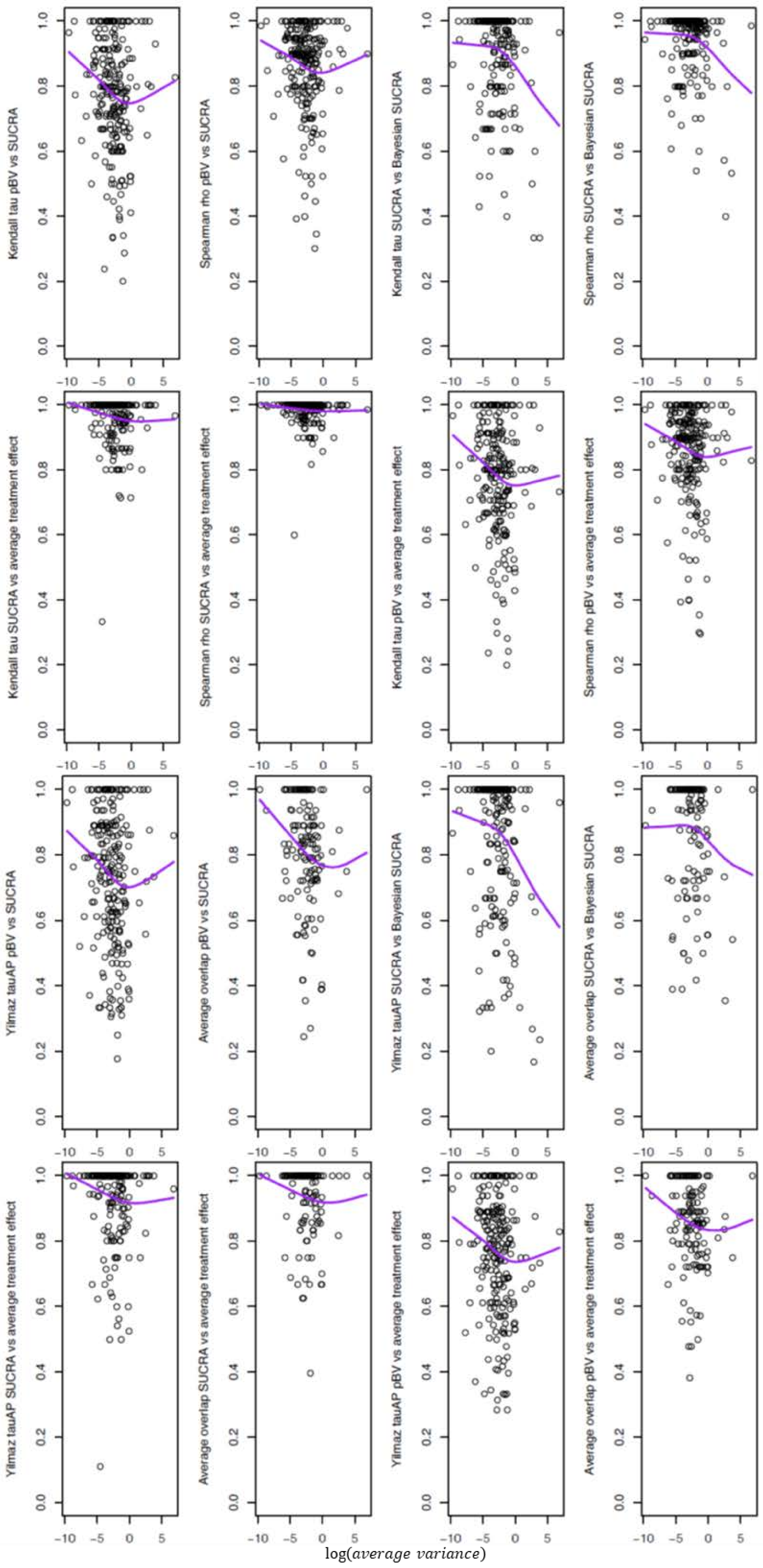

Figure S2: Scatter plots of the average variance in a network and the pairwise agreement between hierarchies from different ranking metrics.

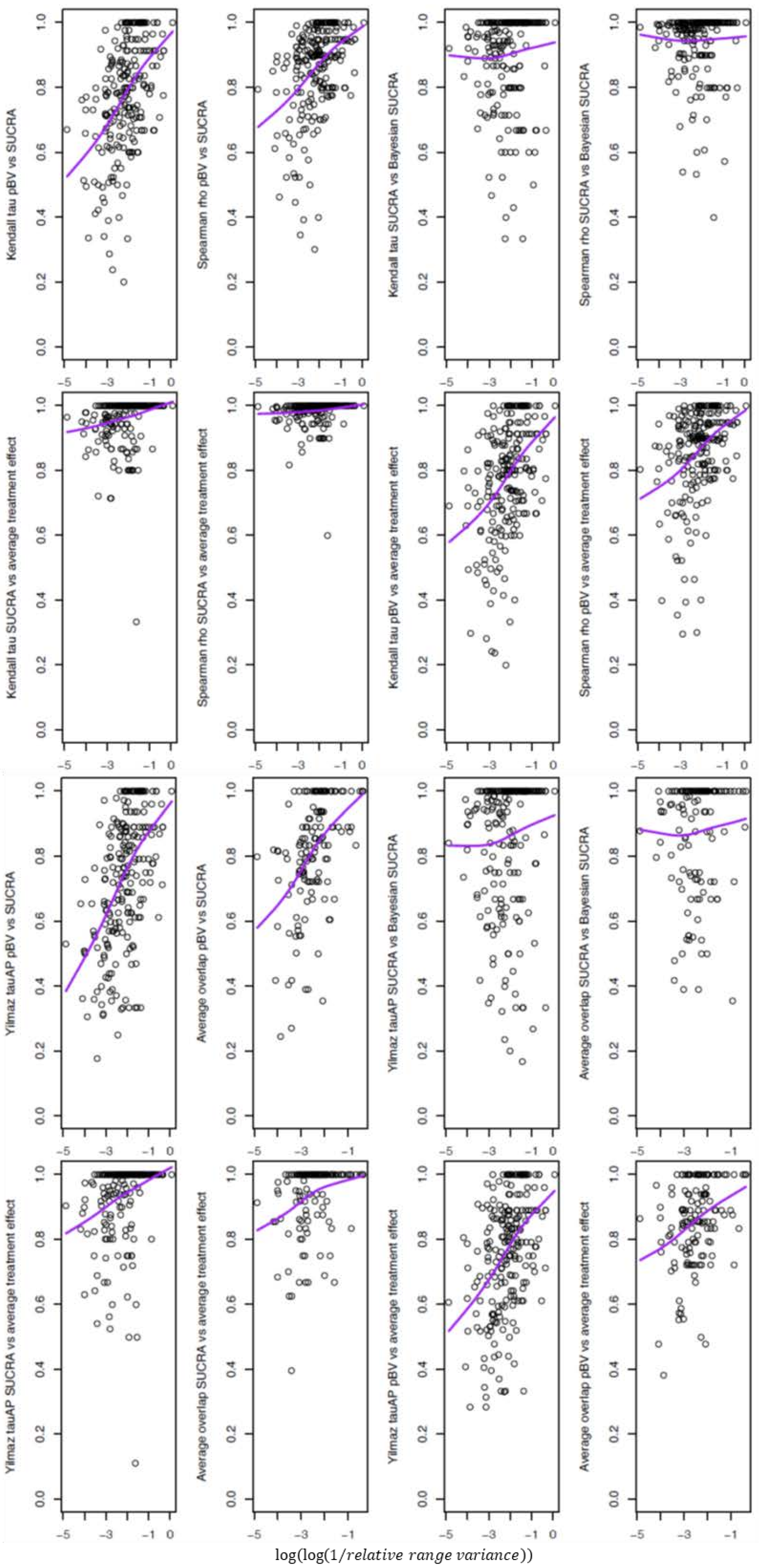

Figure S3: Scatter plots of the relative range of variance in a network and the pairwise agreement between hierarchies from different ranking metrics.

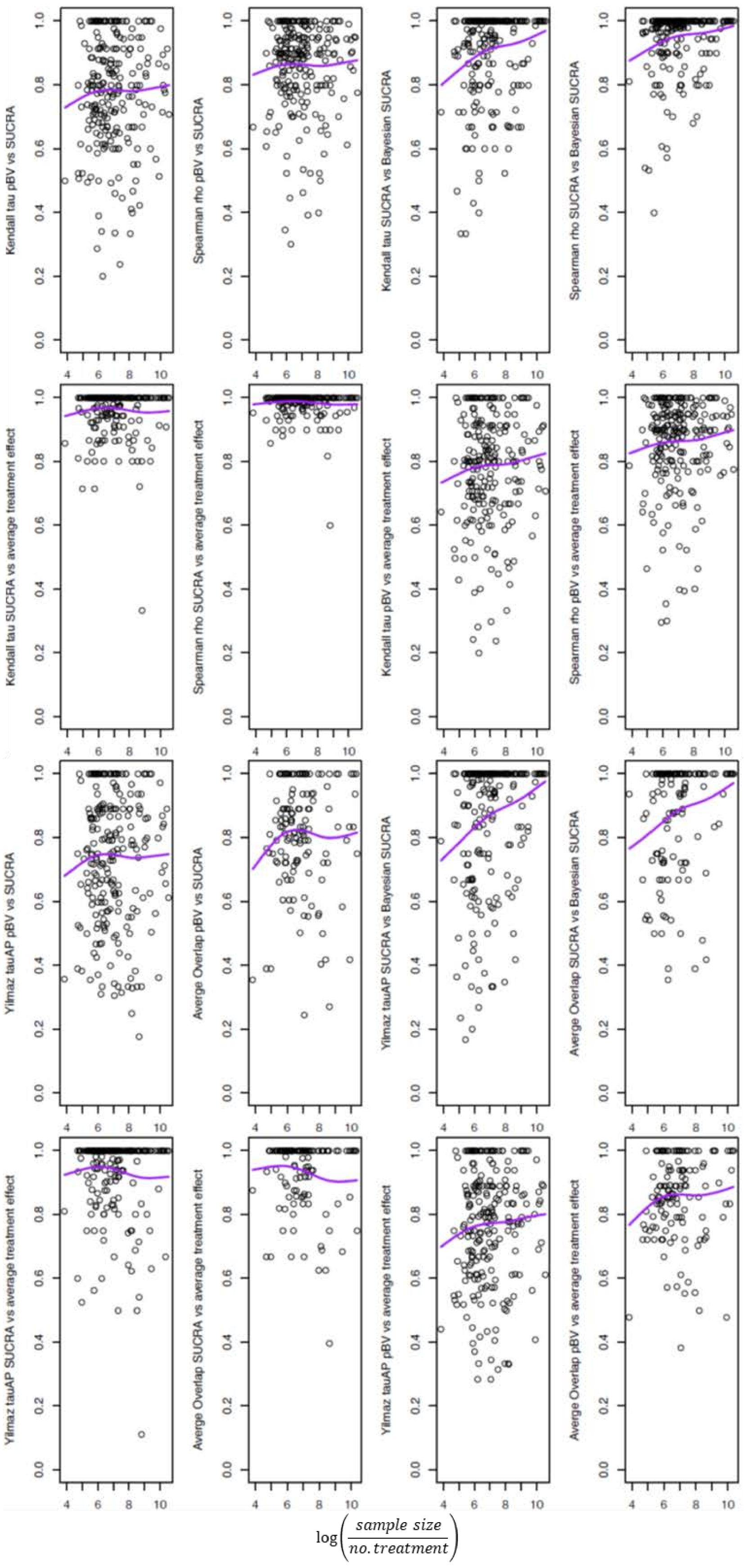

Figure S4: Scatter plots of the total sample size over the number of treatments in a network and the pairwise agreement between hierarchies from different ranking metrics.

Table S1: Pairwise agreement between treatment hierarchies from illustrative network example. *Medians, 1st and 3rd quartiles are reported. p_{BV}: probability of producing the best value; $SUCRA_F$: surface under the cumulative ranking curve; relative treatment effect stands for the relative treatment effect against fictional treatment of average performance. The first three column from the left-hand side present the agreements between rankings obtained using the original data; the equivalent three columns on the right-hand side show the agreements between rankings obtained after reducing the standard error of the Conventional versus ARB treatment effect from 0.7 to a fictional value of 0.01.*

	**Original data**			**Fictional data with increased precision for Conventional treatment versus ARB**		
	p_{BV} **vs** $SUCRA_F$	p_{BV} **vs relative treatment effect**	$SUCRA_F$ **vs relative treatment effect**	p_{BV} **vs** $SUCRA_F$	p_{BV} **vs relative treatment effect**	$SUCRA_F$ **vs relative treatment effect**
**Spearman ρ**	0.64	0.85	0.93	0.89	0.91	1
**Kendall τ**	0.54	0.69	0.87	0.78	0.82	0.99
**Yilmaz τ_{AP}**	0.39	0.44	0.87	0.76	0.81	0.96
**Average Overlap**	0.48	0.54	0.85	0.85	0.94	0.92

APPENDIX – YILMAZ'S τ_{AP} AND AVERAGE OVERLAP

The Yilmaz's τ_{AP} calculates the difference between the probability of observing concordance and the probability of observing discordance between two rankings X and Y, penalising more the discordance between top ranks. It can be computed as

$$\tau_{AP}(X,Y) = \frac{2}{N-1}\sum_{i=2}^{N}\sum_{j<i}\frac{C_{ij}}{i-1} - 1$$

where cij is 1 in case the items i and j are concordant and 0 otherwise; N is the total number of items in the ranking.

As Yilmaz's τ_{AP} is not symmetric, the authors proposed an alternative measure that takes the average between the two τ_{AP}, with the second being the one calculated after swapping the two rankings

$$symm\,\tau_{AP}(X,Y) = \left(\tau_{AP}(X|Y) + \tau_{AP}(Y|X)\right)/2$$

As with the original Kendall's τ, also the Yilmaz's τ_{AP} formula above does not handle ties. Similarly, two formulations to account for this have been proposed[i] and we selected the one that considers correlation as a measure of agreement because more relevant for our purpose. In our chosen version of the Yilmaz's τ_{AP}, the $\tau_{AP,b}$, neither of the two rankings is considered "true and objective" and ties can be present in either or both of them. The formula appears as follows

$$\tau_{AP,b} = \left(\tau_{AP,ties}(X|Y) + \tau_{AP,ties}(Y|X)\right)/2 \qquad \tau_{AP,ties} = \frac{2}{n-t_1}\sum_{i=t_1+1}^{n}\sum_{i<p_i}\frac{C_{ij}}{p_i-1} - 1$$

where t_1 is the number of items tied in position i=1 and pi is the rank of the first item in i's group.

The Average Overlap is a top-weighted measure for top-k rankings that considers the intersection (or overlap) between the two lists, $|X \cap Y|/k$. It calculates the cumulative overlap at increasing depths d, d ∈ {1…k} and average it over the depth (cut-off point) k.

$$AO(X,Y,k) = \frac{1}{k}\sum_{d=1}^{k} A_d \qquad \text{where } A_d = |X \cap Y|/d$$

Unlike the previous measures, the average overlap takes values between 0 and 1.

[i] Julián Urbano and Mónica Marrero, 'The Treatment of Ties in AP Correlation', in *Proceedings of the ACM SIGIR International Conference on Theory of Information Retrieval - ICTIR '17* (the ACM SIGIR International Conference, Amsterdam, The Netherlands: ACM Press, 2017), 321–24, https://doi.org/10.1145/3121050.3121106.
