# Supplementary material for: Agreement between ranking metrics in network meta-analysis: an empirical study
Source: BMJ Open. 2020 Aug 20;10(8):e037744. doi: 10.1136/bmjopen-2020-037744 (PMC7440831; doi:10.1136/bmjopen-2020-037744)
Supplement: Author's manuscript [file bmjopen-2020-037744.draft_revisions.pdf]

BMJ Open

BMJ Open is committed to open peer review. As part of this commitment we make the peer review history of every article we publish publicly available.

When an article is published we post the peer reviewers' comments and the authors' responses online. We also post the versions of the paper that were used during peer review. These are the versions that the peer review comments apply to.

The versions of the paper that follow are the versions that were submitted during the peer review process. They are not the versions of record or the final published versions. They should not be cited or distributed as the published version of this manuscript.

BMJ Open is an open access journal and the full, final, typeset and author-corrected version of record of the manuscript is available on our site with no access controls, subscription charges or pay-per-view fees (http://bmjopen.bmj.com).

If you have any questions on BMJ Open's open peer review process please email info.bmjopen@bmj.com

BMJ Open

Agreement between ranking metrics in network meta-analysis: an empirical study

Journal:	*BMJ Open*
Manuscript ID	bmjopen-2020-037744
Article Type:	Original research
Date Submitted by the Author:	14-Feb-2020
Complete List of Authors:	Chiocchia, Virginia; University of Bern Institute of Social and Preventive Medicine<br>Nikolakopoulou, Adriani; University of Bern Institute of Social and Preventive Medicine<br>Papakonstantinou, Theodoros; University of Bern Institute of Social and Preventive Medicine<br>Egger, Matthias; University of Bern Institute of Social and Preventive Medicine<br>Salanti, Georgia; University of Bern Institute of Social and Preventive Medicine
Keywords:	STATISTICS & RESEARCH METHODS, EPIDEMIOLOGY, PUBLIC HEALTH

SCHOLARONE™
Manuscripts

I, the Submitting Author has the right to grant and does grant on behalf of all authors of the Work (as defined in the below author licence), an exclusive licence and/or a non-exclusive licence for contributions from authors who are: i) UK Crown employees; ii) where BMJ has agreed a CC-BY licence shall apply, and/or iii) in accordance with the terms applicable for US Federal Government officers or employees acting as part of their official duties; on a worldwide, perpetual, irrevocable, royalty-free basis to BMJ Publishing Group Ltd ("BMJ") its licensees and where the relevant Journal is co-owned by BMJ to the co-owners of the Journal, to publish the Work in this journal and any other BMJ products and to exploit all rights, as set out in our licence.

The Submitting Author accepts and understands that any supply made under these terms is made by BMJ to the Submitting Author unless you are acting as an employee on behalf of your employer or a postgraduate student of an affiliated institution which is paying any applicable article publishing charge ("APC") for Open Access articles. Where the Submitting Author wishes to make the Work available on an Open Access basis (and intends to pay the relevant APC), the terms of reuse of such Open Access shall be governed by a Creative Commons licence – details of these licences and which Creative Commons licence will apply to this Work are set out in our licence referred to above.

Other than as permitted in any relevant BMJ Author's Self Archiving Policies, I confirm this Work has not been accepted for publication elsewhere, is not being considered for publication elsewhere and does not duplicate material already published. I confirm all authors consent to publication of this Work and authorise the granting of this licence.

For peer review only

Agreement between ranking metrics in network meta-analysis: an empirical study

Virginia Chiocchia[1], Adriani Nikolakopoulou[1], Theodoros Papakonstantinou[1], Matthias Egger[1], Georgia Salanti[1]

[1] Institute of Social and Preventive Medicine (ISPM), University of Bern, Bern, Switzerland

Correspondence to:

Virginia Chiocchia, Institute of Social and Preventive Medicine, University of Bern, Mittelstrasse 43, CH-3012 Bern, Switzerland.
Email: virginia.chiocchia@ispm.unibe.ch

For peer review only

Abstract 294 words, main text 3004 words, 3 tables, 3 figures, 27 references

Keywords: treatment hierarchy, multiple treatments, evidence synthesis, SUCRA, rank probabilities

For peer review only - http://bmjopen.bmj.com/site/about/guidelines.xhtml

ABSTRACT

Objective

To empirically explore the level of agreement of the treatment hierarchies from different ranking metrics in network meta-analysis (NMA) and to investigate how network characteristics influence the agreement.

Design

Empirical evaluation from re-analysis of network meta-analyses.

Data

232 networks of four or more interventions from randomised controlled trials, published between 1999 and 2015.

Methods

We calculated treatment hierarchies from several ranking metrics: relative treatment effects, probability of producing the best value (p_{BV}) and the surface under the cumulative ranking curve (SUCRA). We estimated the level of agreement between the treatment hierarchies using different measures: Kendall's τ and Spearman's ρ correlation; and the Yilmaz τ_{AP} and Average Overlap, to give more weight to the top of the rankings. Finally, we assessed how the amount of the information present in a network affects the agreement between treatment hierarchies, using the average variance, the relative range of variance, and the total sample size over the number of interventions of a network.

Results

Overall, the pairwise agreement was high for all treatment hierarchies obtained by the different ranking metrics. The highest agreement was observed between SUCRA and the relative treatment effect for both correlation and top-weighted measures whose medians

For peer review only - http://bmjopen.bmj.com/site/about/guidelines.xhtml

were all equal to one. The agreement between rankings decreased for networks with less precise estimates and the hierarchies obtained from p_{BV} appeared to be the most sensitive to large differences in the variance estimates. However, such large differences were rare.

Conclusions

Different ranking metrics address different treatment hierarchy problems, however they produced similar rankings in the published networks. Researchers reporting NMA results can use the ranking metric they prefer, unless there are imprecise estimates or large imbalances in the variance estimates. In this case treatment hierarchies based on both probabilistic and non-probabilistic ranking metrics should be presented.

For peer review only

For peer review only - http://bmjopen.bmj.com/site/about/guidelines.xhtml

STRENGTH AND LIMITATIONS OF THIS STUDY

- To our knowledge, this is the first empirical study exploring the level of agreement of the treatment hierarchies from different ranking metrics in network meta-analysis (NMA).
- The study also explores how agreement is influenced by network characteristics.
- More than 200 published NMAs were re-analysed and three different ranking metrics calculated using both frequentist and Bayesian approaches.
- Other potential factors not investigated in this study could influence the agreement between hierarchies.

For peer review only

INTRODUCTION

Network meta-analysis (NMA) is being increasingly used by policy makers and clinicians to answer one of the key questions in medical decision-making: "what treatment works best for the given condition?" [1,2]. The relative treatment effects, estimated in NMA, can be used to produce ranking metrics: statistical quantities measuring the performance of an intervention on the studied outcomes, thus producing a treatment hierarchy from the most preferable to the least preferable option [3,4].

Despite the importance of treatment hierarchies in evidence-based decision making, various methodological issues related to the ranking metrics have been contested [5–7]. This ongoing methodological debate focuses on the uncertainty and bias in a single ranking metric. Hierarchies produced by different ranking metrics are not expected to agree because ranking metrics differ. For example, a *non-probabilistic ranking metric* such as the treatment effect against a common comparator considers only the mean effect (e.g. the point estimate of the odds-ratio) and ignores the uncertainty with which this is estimated. In contrast, the probability that a treatment achieves a specific rank (a *probabilistic ranking metric*) considers the entire estimated distribution of each treatment effect. However, it is important to understand why and how rankings based on different metrics differ.

There are network characteristics that are expected to influence the agreement of treatment hierarchies from different ranking metrics, such as the precision of the included studies and their distribution across treatment comparisons [4,8]. Larger imbalances in precision in the estimation of the treatment effects affects the agreement of the treatment hierarchies from probabilistic ranking metrics, but it is currently unknown whether in practice these imbalances occur and whether they should inform the choice between different ranking

For peer review only - http://bmjopen.bmj.com/site/about/guidelines.xhtml

metrics. To our knowledge, no empirical studies have explored the level of agreement of treatment hierarchies obtained from different ranking metrics, or examined the network characteristics likely to influence the level of agreement. Here, we empirically evaluated the level of agreement between ranking metrics and examined how the agreement is affected by network features. The article first describes the methods for the calculation of ranking metrics and of specific measures to assess the agreement and to explore factors that affects it, respectively. Then, a network featuring one of the explored factors is shown as an illustrative example to display differences in treatment hierarchies from different ranking metrics. Finally, we present the results from the empirical evaluation and discuss their implications for researchers undertaking network meta-analysis.

METHODS

Data

We re-analysed networks of randomised controlled trials from a database of articles published between 1999 and 2015, including at least 4 treatments; details about the search strategy and inclusion/exclusion criteria can be found in [9,10]. We selected networks reporting arm-level data for binary or continuous outcomes. The database is accessible in the *nmadb* R package [11].

Re-analysis and calculation of ranking metrics

All networks were re-analysed using the relative treatment effect that the original publication used: odds ratio (OR), risk ratio (RR), standardised mean difference (SMD) or mean difference (MD). We estimated relative effects between treatments using a frequentist random-effects NMA model using the *netmeta* R package [12]. For the networks reporting ORs and SMDs we re-analysed them also using Bayesian models using self-programmed NMA routines in JAGS

For peer review only - http://bmjopen.bmj.com/site/about/guidelines.xhtml

(https://github.com/esm-ispm-unibe-ch/NMAJags). To obtain probabilistic ranking metrics in a frequentist setting, we used parametric bootstrap by producing 1000 datasets from the estimated relative effects and their variance-covariance matrix. By averaging over the number of simulated relative effects we derived the *probability of treatment* i *to produce the best value*

$$p_{i,BV} := p_{i,1} = P\left(\mu_{ij} > 0 \;\forall j \in \mathbb{T}\right)$$

where μ_{ij} is the estimated mean relative effect of treatment i against treatment j out of a set $\mathbb{T}$ of T competing treatments. We will refer to this as p_{BV}. This ranking metric indicates how likely a treatment is to produce the largest values for an outcome (or smallest value, if the outcome is harmful). We also calculated the surface under the cumulative ranking curve ($SUCRA^F$) [3]

$$SUCRA_i = \frac{\sum_{r=1}^{T-1} c_{i,r}}{T-1}$$

where $c_{i,r} = \sum_{v=1}^{r} p_{i,v}$ are the cumulative probabilities that treatment i will produce an outcome that is among the r best values (or that it outperforms $T-r$ treatments). SUCRA, unlike p_{BV}, also considers the probability of a treatment to produce unfavourable outcome values. Therefore, the treatment with the largest SUCRA value represents the one that outperforms the competing treatments in the network, meaning that overall it produces preferable outcomes compared to the others. We also obtained SUCRAs within a Bayesian framework ($SUCRA^B$).

To obtain the non-probabilistic ranking metric we fitted an NMA model and estimated related treatment effects. To obtain estimates for all treatments we reparametrize the NMA model so that each treatment is compared to a fictional treatment of average performance [13,14]. The estimated relative effects against a fictional treatment *F* of average efficacy $\hat{\mu}_{iF}$ represent

For peer review only - http://bmjopen.bmj.com/site/about/guidelines.xhtml

the ranking metric and the corresponding hierarchy is obtained simply by ordering the effects from the largest to the smallest (or in ascending order, if the outcome is harmful). The resulting hierarchy is identical to that obtained using relative effects from the conventional NMA model. In the rest of the manuscript, we will refer to this ranking metric simply as relative treatment effect.

Agreement between ranking metrics

To estimate the level of agreement between the treatment hierarchies obtained using the three chosen ranking methods we employed several correlation and similarity measures.

To assess the correlation between ranking metrics we used Kendall's τ [15] and the Spearman's ρ [16]. Both Kendall's τ and Spearman's ρ give the same weight to each item in the ranking. In the context of treatment ranking, the top of the ranking is more important than the bottom. We therefore also used a top-weighted variant of Kendall's τ, Yilmaz τ_{AP} [17], which is based on a probabilistic interpretation of the average precision measure used in information retrieval [18] (see Appendix).

The measures described so far can only be considered for conjoint rankings, i.e. for lists where each item in one list is also present in the other list. Rankings are *non-conjoint* when a ranking is truncated to a certain *depth k* with such lists called *top-k rankings*. We calculated the Average Overlap [19,20], a top-weighted measure for top-k rankings that considers the cumulative intersection (or *overlap*) between the two lists and averages it over a specified depth (cut-off point) *k* (see Appendix for details). We calculated the Average Overlap between pairs of rankings for networks with at least six treatments (139 networks) for a depth *k* equal to half the number of treatments in the network, $k = {}^{T}/_{2}$ (or $((T-1))$ / 2 if *T* is an odd number).

For peer review only - http://bmjopen.bmj.com/site/about/guidelines.xhtml

We calculated the four measures described above to assess the pairwise agreement between the three ranking metrics within the frequentist setting and summarised them for each pair of ranking metrics and each agreement measure using the median and the 1st and 3rd quartiles. The hierarchy according to $SUCRA^B$ was compared to that of its frequentist equivalent to check how often the two disagree.

Influence of network features on the rankings agreement

The main network characteristic considered was the amount of information in the network (reflected in the precision of the estimates). Therefore, for each network we calculated the following measures of information:

- the average variance, calculated as the mean of the variances of the estimated treatment effects $mean(SE^2)$, to show how much information is present in a network altogether;
- the relative range of variance, calculated as $\frac{\max SE^2 - \min SE^2}{\max SE^2}$, to describe differences in information about each intervention within the same networks;
- the total sample size of a network over the number of interventions.

These measures are presented in scatter plots against the agreement measurements for pairs of ranking metrics.

All the codes for the empirical evaluation are available at https://github.com/esm-ispm-unibe-ch/rankingagreement.

ILLUSTRATIVE EXAMPLE

To illustrate the impact of the amount of information on the treatment hierarchies from different ranking metrics, we used a network of nine antihypertensive treatments for primary prevention of cardiovascular disease that presents large differences in the precision of the

For peer review only - http://bmjopen.bmj.com/site/about/guidelines.xhtml

estimates of overall mortality [21]. The network graph and forest plot of relative treatment effects of each treatment versus placebo are presented in **Figure 1**. The relative treatment effects reported are risk ratios (RR) estimated using a random effects NMA model.

Table 1 shows the treatment hierarchies obtained using the three ranking metrics described above. The highest overall agreement is between hierarchies from the $SUCRA^F$ and the relative treatment effect as shown by both correlation (Spearman's ρ = 0.93, Kendall's τ = 0.87) and top-weighted measures (Yilmaz's τ_{AP}= 0.87; Average Overlap = 0.85). The level of agreement decreases when $SUCRA^F$ and the relative treatment effect are compared with p_{BV} rankings (Spearman's ρ = 0.63 and ρ = 0.85 respectively). Agreement with p_{BV} especially decreases when considering top ranks only (Average Overlap is 0.48 for p_{BV} versus $SUCRA^F$ and 0.54 for p_{BV} versus relative treatment effect). All agreement measures are presented in online supplementary **Table S1**.

The reason for this disagreement is explained by the differences in precision in the estimated effects (**Figure 1**). These RRs versus placebo range from 0.82 (Diuretic/Beta-blocker versus placebo) to 0.98 (Beta-blocker versus placebo). All estimates are fairly precise except for the RR of conventional therapy versus placebo whose 95% confidence interval extends from 0.21 to 3.44. This uncertainty in the estimation is due to the fact that conventional therapy is compared only with Angiotensin Receptor Blockers (ARB) via a single study. This large difference in the precision of the estimation of the treatment effects mostly affects the p_{BV} ranking, which disagrees the most with both of the other rankings. Consequently, the Conventional therapy is in the first rank in the p_{BV} hierarchy (because of the large uncertainty) but only features in the third/fourth and sixth rank using the relative treatment effects and $SUCRA^F$ hierarchies, respectively.

For peer review only - http://bmjopen.bmj.com/site/about/guidelines.xhtml

To explore how the hierarchies for this network would change in case of increased precision, we reduced the standard error of the Conventional versus ARB treatment effect from the original 0.7 to a fictional value of 0.01 resulting in a confidence interval 0.77 to 0.96. The columns in the right-hand side of **Table 1** display the three equivalent rankings after the standard error reduction. The conventional treatment has moved up in the hierarchy according to $SUCRA^F$ and moved down in the one based on p_{BV}, as expected. The treatment hierarchies obtained from the $SUCRA^F$ and the relative treatment effect are now identical (Conventional and ARB share the 3.5 rank because they have the same effect estimate) and the agreement with the p_{BV} rankings also improved (p_{BV} versus $SUCRA^F$ Spearman's ρ = 0.89, Average Overlap = 0.85; p_{BV} versus relative treatment effect Spearman's ρ = 0.91, Average Overlap = 0.94; online supplementary **Table S1**).

RESULTS

A total of 232 networks were included in our dataset. Their characteristics are shown in **Table 2**. The majority of networks (133 NMAs, 57.3%) did not report any ranking metrics in the original publication. Among those which used a ranking metric to produce a treatment hierarchy, the probability of being the best was the most popular metric followed by the SUCRA with 35.8% and 6.9% of networks reporting them, respectively.

Table 3 presents the medians and quartiles for each similarity measures. All hierarchies showed a high level of pairwise agreement, although the hierarchies obtained from the $SUCRA^F$ and the relative treatment effect presented the highest values for both unweighted and with top-weighted measures (all measures' median equals 1). Only 4 networks (less than 2%) had a Spearman's correlation between $SUCRA^F$ and the relative treatment effect less than 90% (not reported). The correlation becomes less between the p_{BV} rankings and those

For peer review only - http://bmjopen.bmj.com/site/about/guidelines.xhtml

obtained from the other two ranking metrics with Spearman's ρ median decreasing to 0.9 and Kendall's τ decreasing to 0.8. The Spearman's correlation between these rankings was less than 90% in about 50% of the networks (in 116 and 111 networks for p_{BV} versus $SUCRA^F$ and p_{BV} versus relative effect, respectively; results not reported). The pairwise agreement between the p_{BV} rankings and the other rankings also decreased when considering only top ranks (p_{BV} versus $SUCRA^F$ Yilmaz's τ_{AP} = 0.77, Average Overlap = 0.83; p_{BV} versus relative treatment effect Yilmaz's τ_{AP} = 0.79, Average Overlap = 0.88).

The SUCRAs from frequentist and Bayesian settings ($SUCRA^F$ and $SUCRA^B$) were compared in 126 networks (82 networks using the Average Overlap measure) as these reported OR and SMD as original measures. The relevant rankings do not differ much as shown by the median values of the agreement measures all equal to 1 and their narrow interquartile ranges (**Table 3**). Nevertheless, a few networks showed a much lower agreement between the two SUCRAs. These networks provide posterior effect estimates for which the Normal approximation is not optimal. Such cases were however uncommon as in only 6% of the networks the Spearman's correlation between $SUCRA^F$ and $SUCRA^B$ was less than 90%. Plots for the Normal distributions from the frequentist setting and the posterior distributions of the log odds-ratios (LOR) for a network with a Spearman's ρ of 0.6 between the two SUCRAs is available in online supplementary **Figure S1** [22].

Figure 2 presents how Spearman's ρ and the Average Overlap vary with the average variance of the relative treatment effect estimates in a network (scatter plots for the Kendall's τ and the Yilmaz's τ_{AP} are available in online supplementary **Figure S2**). The treatment hierarchies agree more in networks with more precise estimates (left hand side of the plots).

The association between Spearman's ρ or Average Overlap and the relative range of variance in a network (here transformed to a double logarithm of the inverse values) are displayed in

For peer review only - http://bmjopen.bmj.com/site/about/guidelines.xhtml

For peer review only

Figure 3. On the right-hand side of each plot we can find networks with smaller differences in the precision of the treatment effect estimates. Treatment hierarchies for these networks show a larger agreement than for those with larger differences in precision. The plots of the impact of the relative range of variance on all measures are available in online supplementary **Figure S3**.

The total sample size in a network over the number of interventions has a similar impact on the level of agreement between hierarchies. This confirms that the agreement between hierarchies increases for networks with a large total sample size compared to the number of treatments and, more generally, it increases with the amount of information present in a network (online supplementary **Figure S4**).

DISCUSSION

Our empirical evaluation showed that in practice the level of agreement between treatment hierarchies is overall high for all ranking metrics used. The agreement between treatment hierarchies from $SUCRA$ and relative treatment effect was very often perfect. The agreement between the rankings from $SUCRA$ or relative treatment effect and the ranking from p_{BV} was good but decreased when the top-ranked interventions are of interest. The agreement is higher for networks with precise estimates and small imbalances in precision.

Several factors can be responsible for imprecision in the estimation of the relative treatment effects in a network:

- large sampling error, determined by a small sample size, small number of events or a large standard deviation;
- poor connectivity of the network, when only a few links and few closed loops of evidence connect the treatments;

For peer review only - http://bmjopen.bmj.com/site/about/guidelines.xhtml

- residual inconsistency;
- heterogeneity in the relative treatment effects.

Random-effects models tend to provide relative treatment effects with similar precision as heterogeneity increases. In contrast, in the absence of heterogeneity when fixed-effects models are used, the precision of the effects can vary a lot according to the amount of data available for each intervention. In the latter case, the ranking metrics are likely to disagree.

Our results also confirm that a treatment hierarchy can differ when the uncertainty in the estimation is incorporated into the ranking metric [8,23] and that rankings from the p_{BV} seem to be the most sensitive to differences in precision in the estimation of treatment effects. We showed graphically that the agreement is less in networks with more uncertainty and with larger imbalances in the variance estimates. However, we also found that such large imbalances do not occur frequently in real data and in the majority of cases the different treatment hierarchies have a relatively high agreement.

We acknowledge that there could be other factors influencing the agreement between hierarchies that we did not explore, such as the risk of bias [23,24] and the chosen effect measures [25]. However, we think it is unlikely that such features play a big role in ranking agreement unless assumptions are violated or data in the network is sparse [26].

To our knowledge, this is the first empirical study assessing the level of agreement between treatment hierarchies from ranking metrics in NMA and it provides further insights into the properties of the different methods. In this context, it is important to stress that neither the objective nor the findings of this empirical evaluation imply that a hierarchy for a particular metric works better or is more accurate than one obtained from another ranking metric. The reason why this sort of comparison cannot be made is that each ranking metric address a specific treatment hierarchy problem. For example, the $SUCRA$ ranking addresses the issue

For peer review only - http://bmjopen.bmj.com/site/about/guidelines.xhtml

of which treatment outperforms most of the competing interventions, while the ranking based on the relative treatment effect gives an answer to the problem of which treatment is associated with the largest average effect for the outcome considered.

Our study shows that, despite theoretical differences between ranking metrics and some extreme examples, they produce very similar treatment hierarchies in published networks. In networks with large amount of data for each treatment, hierarchies based on SUCRA or the relative treatment effect will almost always agree. Large imbalances in the precision of the treatment effect estimates do not occur often enough to motivate a choice between the different ranking metrics. Therefore, our advice to researchers presenting results from NMA is the following: *if the NMA estimated effects are precise,* to use the ranking metric they prefer; *if at least one NMA estimated effect is imprecise,* to refrain from making bold statements about treatment hierarchy and present hierarchies from both probabilistic (e.g. SUCRA or rank probabilities) and non-probabilistic metrics (e.g. relative treatments effects).

Author contributions

VC designed the study, analysed the data, interpreted the results of the empirical evaluation, and drafted the manuscript. GS designed the study, interpreted the results of the empirical evaluation and revised the manuscript. AN provided input into the study design and the data analysis, interpreted the results of the empirical evaluation and revised the manuscript. TP developed and manages the database where networks' data was accessed, provided input into the data analysis and revised the manuscript. ME provided input into the study design and revised the manuscript. All the authors approved the final version of the submitted manuscript.

For peer review only - http://bmjopen.bmj.com/site/about/guidelines.xhtml

Funding

This work was supported by the Swiss National Science Foundation grant/award number 179158.

Competing Interests

All authors have completed the ICMJE uniform disclosure form and declare: all authors had financial support from the Swiss National Science Foundation for the submitted work; no financial relationships with any organizations that might have an interest in the submitted work in the previous three years; no other relationships or activities that could appear to have influenced the submitted work.

Patient consent for publication

Not required.

Data sharing statement

The data for the network meta-analyses included in this study are available in the database accessible using the *nmadb* R package [11].

For peer review only

For peer review only - http://bmjopen.bmj.com/site/about/guidelines.xhtml

References

1 Efthimiou O, Debray TPA, van Valkenhoef G, *et al.* GetReal in network meta-analysis: a review of the methodology: reviewNMA. *Res Synth Methods* 2016;**7**:236–63. doi:10.1002/jrsm.1195

2 Del Fiol G, Workman TE, Gorman PN. Clinical questions raised by clinicians at the point of care: a systematic review. *JAMA Intern Med* 2014;**174**:710–8. doi:10.1001/jamainternmed.2014.368

3 Salanti G, Ades AE, Ioannidis JPA. Graphical methods and numerical summaries for presenting results from multiple-treatment meta-analysis: an overview and tutorial. *J Clin Epidemiol* 2011;**64**:163–71. doi:10.1016/j.jclinepi.2010.03.016

4 Rücker G, Schwarzer G. Ranking treatments in frequentist network meta-analysis works without resampling methods. *BMC Med Res Methodol* 2015;**15**:58. doi:10.1186/s12874-015-0060-8

5 Trinquart L, Attiche N, Bafeta A, *et al.* Uncertainty in Treatment Rankings: Reanalysis of Network Meta-analyses of Randomized Trials. *Ann Intern Med* 2016;**164**:666–73. doi:10.7326/M15-2521

6 Kibret T, Richer D, Beyene J. Bias in identification of the best treatment in a Bayesian network meta-analysis for binary outcome: a simulation study. *Clin Epidemiol* 2014;**6**:451–60. doi:10.2147/CLEP.S69660

7 Veroniki AA, Straus SE, Rücker G, *et al.* Is providing uncertainty intervals in treatment ranking helpful in a network meta-analysis? *J Clin Epidemiol* 2018;**100**:122–9. doi:10.1016/j.jclinepi.2018.02.009

8 Jansen JP, Trikalinos T, Cappelleri JC, *et al.* Indirect Treatment Comparison/Network Meta-Analysis Study Questionnaire to Assess Relevance and Credibility to Inform Health Care Decision Making: An ISPOR-AMCP-NPC Good Practice Task Force Report. *Value Health* 2014;**17**:157–73. doi:10.1016/j.jval.2014.01.004

9 Petropoulou M, Nikolakopoulou A, Veroniki A-A, *et al.* Bibliographic study showed improving statistical methodology of network meta-analyses published between 1999 and 2015. *J Clin Epidemiol* 2017;**82**:20–8. doi:10.1016/j.jclinepi.2016.11.002

10 Nikolakopoulou A, Chaimani A, Veroniki AA, *et al.* Characteristics of networks of interventions: a description of a database of 186 published networks. *PloS One* 2014;**9**:e86754. doi:10.1371/journal.pone.0086754

11 Papakonstantinou T. *nmadb: Network Meta-Analysis Database API.* 2019. https://CRAN.R-project.org/package=nmadb

12 Rücker G, Krahn U, König J, *et al.* *netmeta: Network Meta-Analysis using Frequentist Methods.* 2019. https://github.com/guido-s/netmeta http://meta-analysis-with-r.org.

13 Hosmer DW, Lemeshow S. *Applied Logistic Regression: Hosmer/Applied Logistic Regression*. Hoboken, NJ, USA: : John Wiley & Sons, Inc. 2000. doi:10.1002/0471722146

14 Nikolakopoulou A, Mavridis D, Chiocchia V, *et al.* PreTA: A network meta-analysis ranking metric measuring the probability of being preferable than the average treatment. *Res Synth Methods* (submitted).

15 Kendall MG. THE TREATMENT OF TIES IN RANKING PROBLEMS. *Biometrika* 1945;**33**:239–51. doi:10.1093/biomet/33.3.239

16 Spearman C. The Proof and Measurement of Association between Two Things. *Am J Psychol* 1904;**15**:72. doi:10.2307/1412159

17 Yilmaz E, Aslam JA, Robertson S. A new rank correlation coefficient for information retrieval. In: *Proceedings of the 31st annual international ACM SIGIR conference on Research and development in information retrieval - SIGIR '08*. Singapore, Singapore: : ACM Press 2008. 587. doi:10.1145/1390334.1390435

For peer review only - http://bmjopen.bmj.com/site/about/guidelines.xhtml

18 Yilmaz E, Aslam JA. Estimating Average Precision with Incomplete and Imperfect Judgments. In: *Proceedings of the 15th ACM International Conference on Information and Knowledge Management*. New York, NY, USA: : ACM 2006. 102–111. doi:10.1145/1183614.1183633

19 Fagin R, Kumar R, Sivakumar D. Comparing Top K Lists. In: *Proceedings of the Fourteenth Annual ACM-SIAM Symposium on Discrete Algorithms*. Philadelphia, PA, USA: : Society for Industrial and Applied Mathematics 2003. 28–36.http://dl.acm.org/citation.cfm?id=644108.644113 (accessed 15 May 2019).

20 Wu S, Crestani F. Methods for Ranking Information Retrieval Systems Without Relevance Judgments. In: *Proceedings of the 2003 ACM Symposium on Applied Computing*. New York, NY, USA: : ACM 2003. 811–816. doi:10.1145/952532.952693

21 Fretheim A, Odgaard-Jensen J, Brørs O, *et al.* Comparative effectiveness of antihypertensive medication for primary prevention of cardiovascular disease: systematic review and multiple treatments meta-analysis. *BMC Med* 2012;**10**:33. doi:10.1186/1741-7015-10-33

22 Greco T, Calabrò MG, Covello RD, *et al.* A Bayesian network meta-analysis on the effect of inodilatory agents on mortality. *Br J Anaesth* 2015;**114**:746–56. doi:10.1093/bja/aeu446

23 Chaimani A, Vasiliadis HS, Pandis N, *et al.* Effects of study precision and risk of bias in networks of interventions: a network meta-epidemiological study. *Int J Epidemiol* 2013;**42**:1120–31. doi:10.1093/ije/dyt074

24 Trinquart L, Abbé A, Ravaud P. Impact of Reporting Bias in Network Meta-Analysis of Antidepressant Placebo-Controlled Trials. *PLoS ONE* 2012;**7**:e35219. doi:10.1371/journal.pone.0035219

25 Norton EC, Miller MM, Wang JJ, *et al.* Rank Reversal in Indirect Comparisons. *Value Health* 2012;**15**:1137–40. doi:10.1016/j.jval.2012.06.001

26 van Valkenhoef G, Ades AE. Evidence Synthesis Assumes Additivity on the Scale of Measurement: Response to "Rank Reversal in Indirect Comparisons" by Norton et al. *Value Health* 2013;**16**:449–51. doi:10.1016/j.jval.2012.11.012

27 Urbano J, Marrero M. The Treatment of Ties in AP Correlation. In: *Proceedings of the ACM SIGIR International Conference on Theory of Information Retrieval - ICTIR '17*. Amsterdam, The Netherlands: : ACM Press 2017. 321–4. doi:10.1145/3121050.3121106

For peer review only

For peer review only - http://bmjopen.bmj.com/site/about/guidelines.xhtml

Table 1: Example of treatment hierarchies from different ranking metrics for a network of nine antihypertensive treatment for primary prevention of cardiovascular disease [21].

Treatment	Original data			Fictional data with increased precision for Conventional treatment versus ARB		
	p_{BV} ranks	$SUCRA_F$ ranks	Relative treatment effect ranks	p_{BV} ranks	$SUCRA_F$ ranks	Relative treatment effect ranks
**Conventional**	1	6	3.5	3	4	3.5
**Diuretic/Beta-blocker**	2	1	1	1	1	1
**ARB**	3	3	3.5	4.5	3	3.5
**CCB**	4	2	2	2	2	2
**Alpha-blocker**	5	7	7	4.5	7	7
**ACE-inhibitor**	6	4	5	6.5	5	5
**Diuretic**	7	5	6	6.5	6	6
**Placebo**	8.5	9	9	8.5	9	9
**Beta-Blocker**	8.5	8	8	8.5	8	8

ACE=Angiotensin Converting Enzyme; CCB=Calcium Channel Blockers; ARB=Angiotensin Receptor Blockers. p_{BV}: probability of producing the best value; $SUCRA_F$: surface under the cumulative ranking curve (calculated in frequentist setting); relative treatment effect stands for the relative treatment effect against fictional treatment of average performance. The first three rankings from the left-hand side are obtained using the original data; the equivalent three rankings on the right-hand side are produced by reducing the standard error of the Conventional versus ARB treatment effect from 0.7 to a fictional value of 0.01.

Table 2: Characteristics of the 232 NMAs included in the re-analysis.

Characteristics of networks	Median	IQR
Median number of treatments compared	6	(5, 9)
Median number of studies included	19	(12, 34)
Median total sample size	6100	(2514, 17264)
	Number of NMAs	%
Beneficial outcome	97	41.8%
Dichotomous outcome	185	79.7%
Continuous outcome	47	20.3%
Published before 2010	42	18.1%
Ranking metric used in original publication (non-exclusive):		
Probability of producing the best value	83	35.8%
Rankograms	7	3%
Median or mean rank	3	1.3%
SUCRA	16	6.9%
Other	2	0.9%
None	133	57.3%

For peer review only - http://bmjopen.bmj.com/site/about/guidelines.xhtml

**Published in general medicine journals†**	125	53.9%
**Published in health services research journals‡**	3	1.3%
**Published in specialty journals**	104	44.8%

IQR: interquartile range; NMA: network meta-analysis; SUCRA: surface under the cumulative ranking curve.
† Includes the categories Medicine, General & Internal, Pharmacology & Pharmacy, Research & Experimental, Primary Health Care.
‡ Includes the categories Health Care Sciences & Services, Health Policy & Services.

Table 3: Pairwise agreement between treatment hierarchies obtained from the different ranking metrics measured by Spearman ρ, Kendall τ, Yilmaz τ_{AP} and Average Overlap.

	p_{BV} vs $SUCRA_F$	$SUCRA_F$ vs relative treatment effect	p_{BV} vs relative treatment effect	$SUCRA_F$ vs $SUCRA_B$
**Spearman ρ**	0.9 (0.8, 0.96)	1 (0.99, 1)	0.9 (0.8, 0.97)	1 (0.98, 1)
**Kendall τ**	0.8 (0.67, 0.91)	1 (0.95, 1)	0.8 (0.69, 0.91)	1 (0.93, 1)
**Yilmaz τ_{AP}**	0.78 (0.6, 0.9)	1 (0.93, 1)	0.79 (0.65, 0.9)	1 (0.93, 1)
**Average Overlap**	0.85 (0.72, 0.96)	1 (0.91, 1)	0.88 (0.79, 1)	1 (0.94, 1)

Medians, 1st and 3rd quartiles are reported. p_{BV}: probability of producing the best value; $SUCRA_F$: surface under the cumulative ranking curve (calculated in frequentist setting); $SUCRA_B$: surface under the cumulative ranking curve (calculated in Bayesian setting); relative treatment effect stands for the relative treatment effect against fictional treatment of average performance.

Figure 1: (left panel) Network graph of network of nine antihypertensive treatments for primary prevention of cardiovascular disease *[21]**.** Line width is proportional to inverse standard error of random effects model comparing two treatments.* ***(right panel) Forest plots of relative treatment effects of overall mortality for each treatment versus placebo.*** *RR: risk ratio; ACE=Angiotensin Converting Enzyme; CCB=Calcium Channel Blockers; ARB=Angiotensin Receptor Blockers; SE=standard error.*

Figure 2: Scatter plots of the average variance in a network and the pairwise agreement between hierarchies from different ranking metrics. *The average variance is calculated as the mean of the variances of the estimated treatment effects and describes the average information present in a network. More imprecise network are on the right-hand side of the plots. Spearman ρ (top row) and Average Overlap (bottom row) values for the pairwise agreement between p_{BV} and SUCRA (first column), SUCRA and relative treatment effect (second column), p_{BV} and relative treatment effect (third column). Purple line: cubic smoothing spline with five degrees of freedom.*

Figure 3: Scatter plots of the relative range of variance in a network and the pairwise agreement between hierarchies from different ranking metrics. *The relative range of variance, calculated as $\frac{max\,SE^2 - min\,SE^2}{max\,SE^2}$, indicates how much the information differs between interventions in the same networks. Networks with larger differences in variance are on the left-hand side of the plots. Spearman ρ (top row) and Average Overlap (bottom row) values for the pairwise agreement between p_{BV} and SUCRA (first column), SUCRA and relative treatment effect (second column), p_{BV} and relative treatment effect (third column). Purple line: cubic smoothing spline with five degrees of freedom.*

For peer review only - http://bmjopen.bmj.com/site/about/guidelines.xhtml

APPENDIX

The Yilmaz's τ_{AP} calculates the difference between the probability of observing concordance and the probability of observing discordance between two rankings X and Y, penalising more the discordance between top ranks. It can be computed as

$$\tau_{AP}(X,Y) = \frac{2}{N-1}\sum_{i=2}^{N}\sum_{j<i}\frac{C_{ij}}{i-1} - 1$$

where c_{ij} is 1 in case the items *i* and *j* are concordant and 0 otherwise; *N* is the total number of items in the ranking.

As Yilmaz's τ_{AP} is not symmetric, the authors proposed an alternative measure that takes the average between the two τ_{AP}, with the second being the one calculated after swapping the two rankings

$$symm\,\tau_{AP}(X,Y) = \left(\tau_{AP}(X|Y) + \tau_{AP}(Y|X)\right)/2$$

As with the original Kendall's τ, also the Yilmaz's τ_{AP} formula above does not handle ties. Similarly, two formulations to account for this have been proposed [27] and we selected the one that considers correlation as a measure of agreement because more relevant for our purpose. In our chosen version of the Yilmaz's τ_{AP}, the $\tau_{AP,b}$, neither of the two rankings is considered "true and objective" and ties can be present in either or both of them. The formula appears as follows

$$\tau_{AP,b} = \left(\tau_{AP,ties}(X|Y) + \tau_{AP,ties}(Y|X)\right)/2 \qquad \tau_{AP,ties} = \frac{2}{n-t_1}\sum_{i=t_1+1}^{n}\sum_{i<p_i}\frac{C_{ij}}{p_i-1} - 1$$

where t_1 is the number of items tied in position *i*=1 and p_i is the rank of the first item in *i*'s group.

The Average Overlap is a top-weighted measure for top-k rankings that considers the intersection (or *overlap*) between the two lists, $|X \cap Y|/k$. It calculates the cumulative

For peer review only - http://bmjopen.bmj.com/site/about/guidelines.xhtml

overlap at increasing depths *d*, $d \in \{1 \ldots k\}$ and average it over the depth (cut-off point) *k*.

$$AO(X,Y,k) = \frac{1}{k}\sum_{d=1}^{k} A_d \qquad \text{where } A_d = |X \cap Y|/d$$

Unlike the previous measures, the average overlap takes values between 0 and 1.

For peer review only

For peer review only - http://bmjopen.bmj.com/site/about/guidelines.xhtml

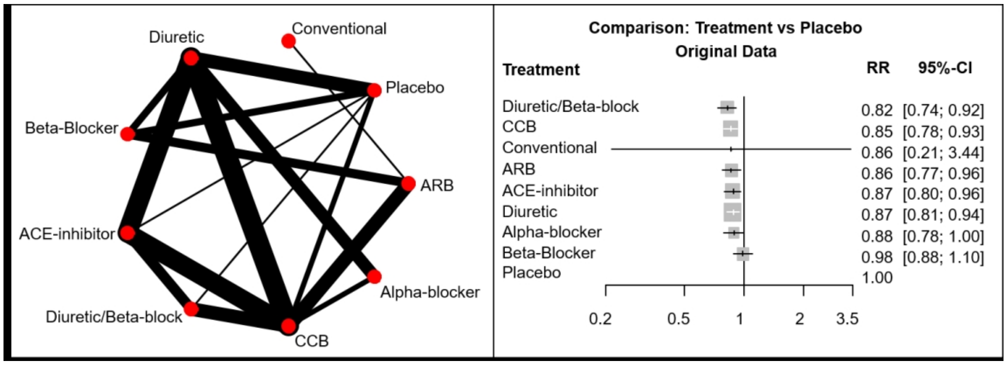

(left panel) Network graph of network of nine antihypertensive treatments for primary prevention of cardiovascular disease [21]. Line width is proportional to inverse standard error of random effects model comparing two treatments. **(right panel) Forest plots of relative treatment effects of overall mortality for each treatment versus placebo.** RR: risk ratio; ACE=Angiotensin Converting Enzyme; CCB=Calcium Channel Blockers; ARB=Angiotensin Receptor Blockers; SE=standard error.

258x92mm (150 x 150 DPI)

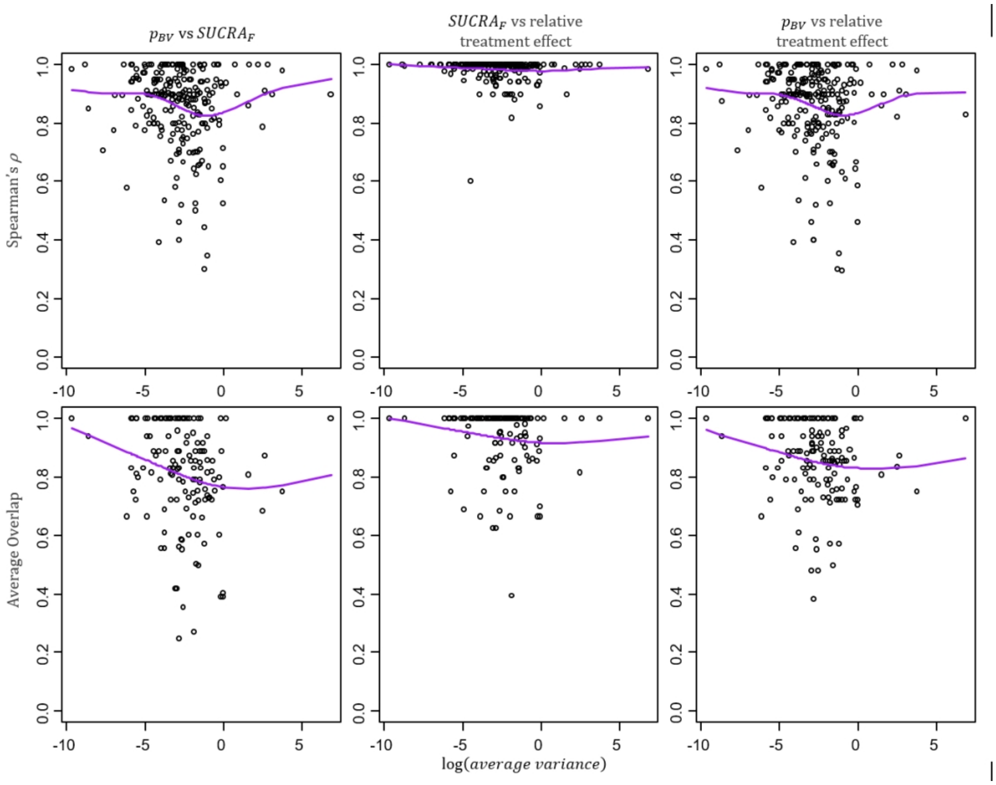

Scatter plots of the average variance in a network and the pairwise agreement between hierarchies from different ranking metrics. The average variance is calculated as the mean of the variances of the estimated treatment effects and describes the average information present in a network. More imprecise network are on the right-hand side of the plots. Spearman ρ (top row) and Average Overlap (bottom row) values for the pairwise agreement between p_{BV} and SUCRA (first column), SUCRA and relative treatment effect (second column), p_{BV} and relative treatment effect (third column). Purple line: cubic smoothing spline with five degrees of freedom.

227x176mm (150 x 150 DPI)

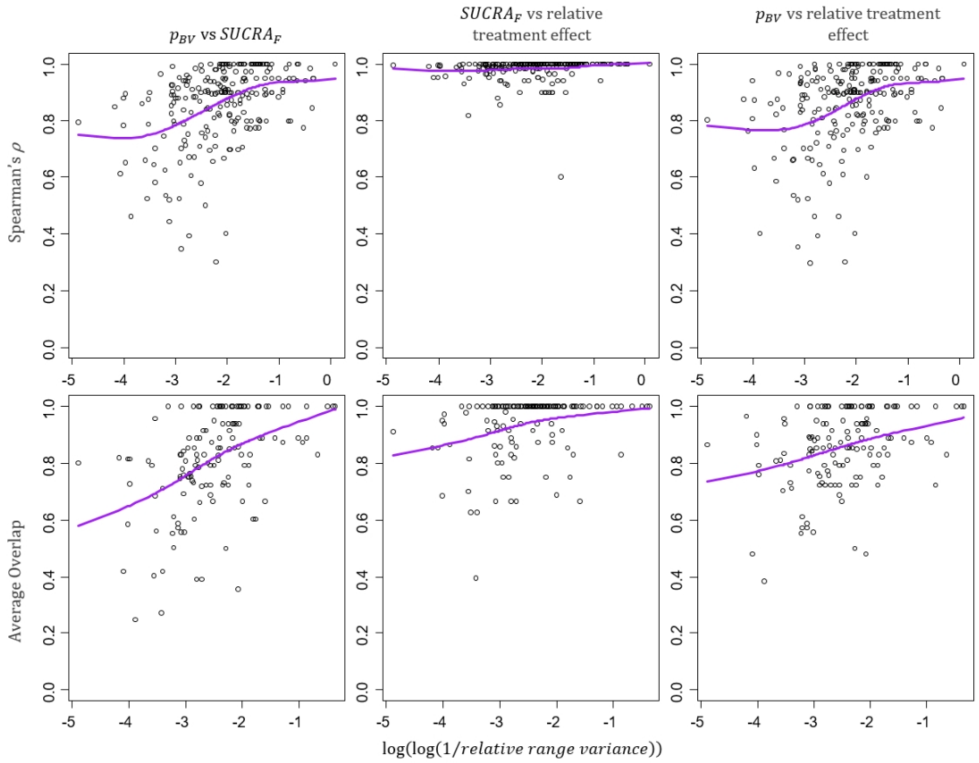

[redacted]

Figure 3: Scatter plots of the relative range of variance in a network and the pairwise agreement between hierarchies from different ranking metrics. The relative range of variance, calculated as $(\max(SE^2) - \min(SE^2)) / \max(SE^2)$, indicates how much the information differs between interventions in the same networks. Networks with larger differences in variance are on the left-hand side of the plots. Spearman ρ (top row) and Average Overlap (bottom row) values for the pairwise agreement between p_{BV} and SUCRA (first column), SUCRA and relative treatment effect (second column), p_{BV} and relative treatment effect (third column). Purple line: cubic smoothing spline with five degrees of freedom.

214x176mm (150 x 150 DPI)

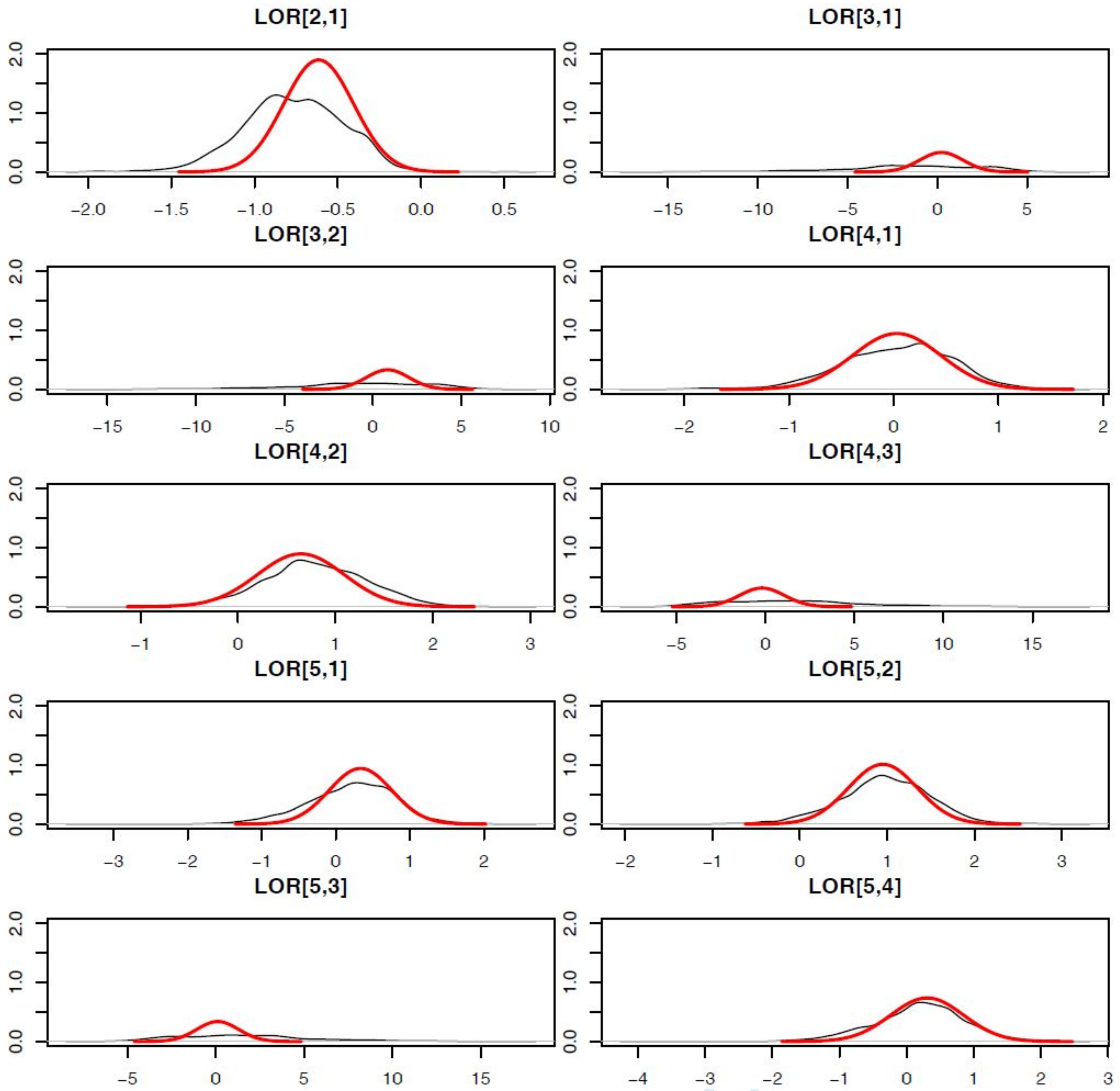

Figure S1: Normal distributions of relative treatment effect estimates from frequentist setting (red line) superimposed on posterior distributions of the relative treatment effects from Bayesian model (black line) for a network with Spearman's ρ of 0.6 between $SUCRA_F$ and $SUCRA_B$. The network meta-analysis used analysed the effects of four inodilators and placebo on survival in adult cardiac surgery patients (Greco et al., Br J Anaesth 2015). LOR = log odds ratios; number in square brackets represents the different interventions in the network.

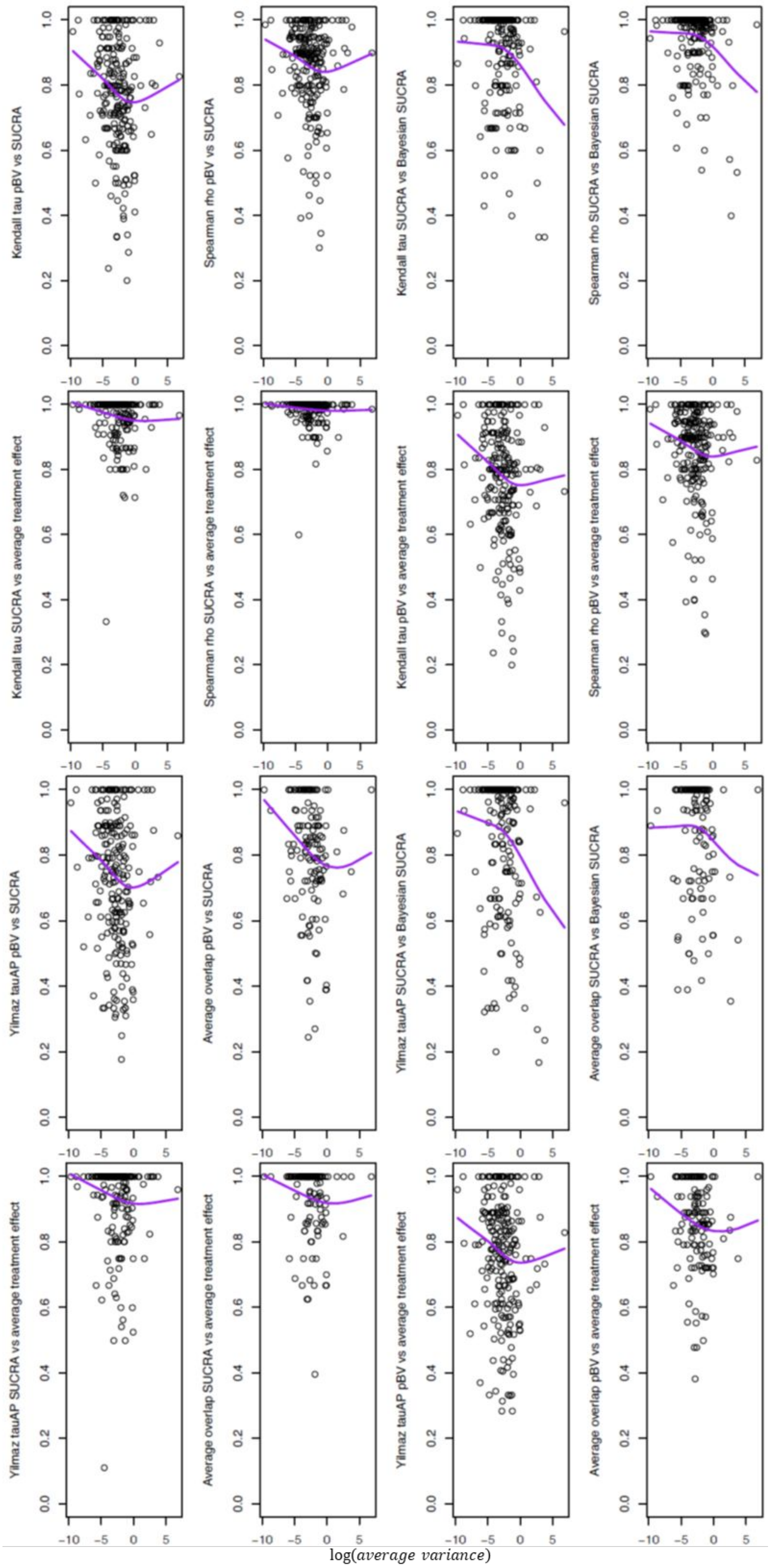

Figure S2: Scatter plots of the average variance in a network and the pairwise agreement between hierarchies from different ranking metrics.

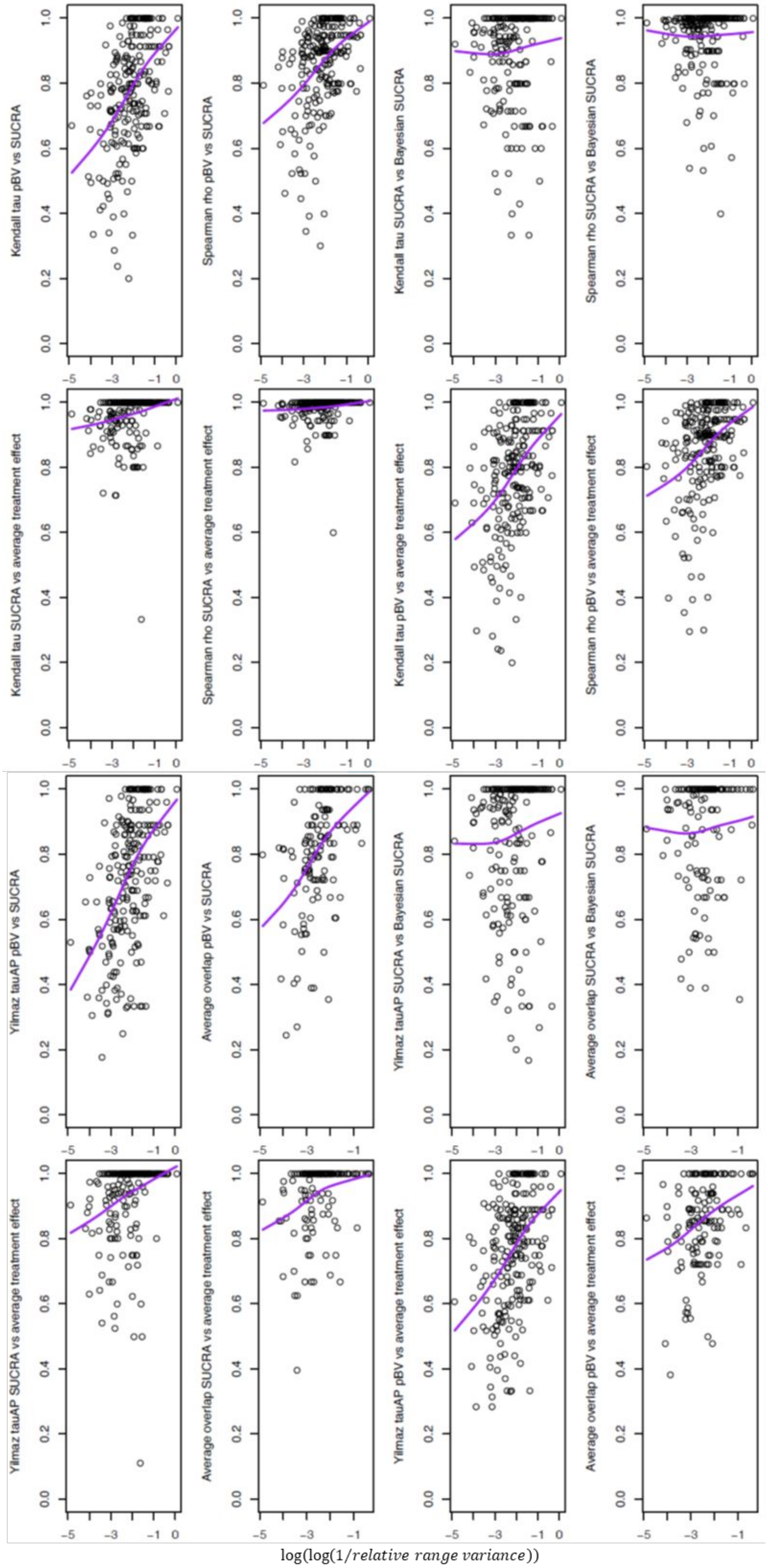

Figure S3: Scatter plots of the relative range of variance in a network and the pairwise agreement between hierarchies from different ranking metrics.

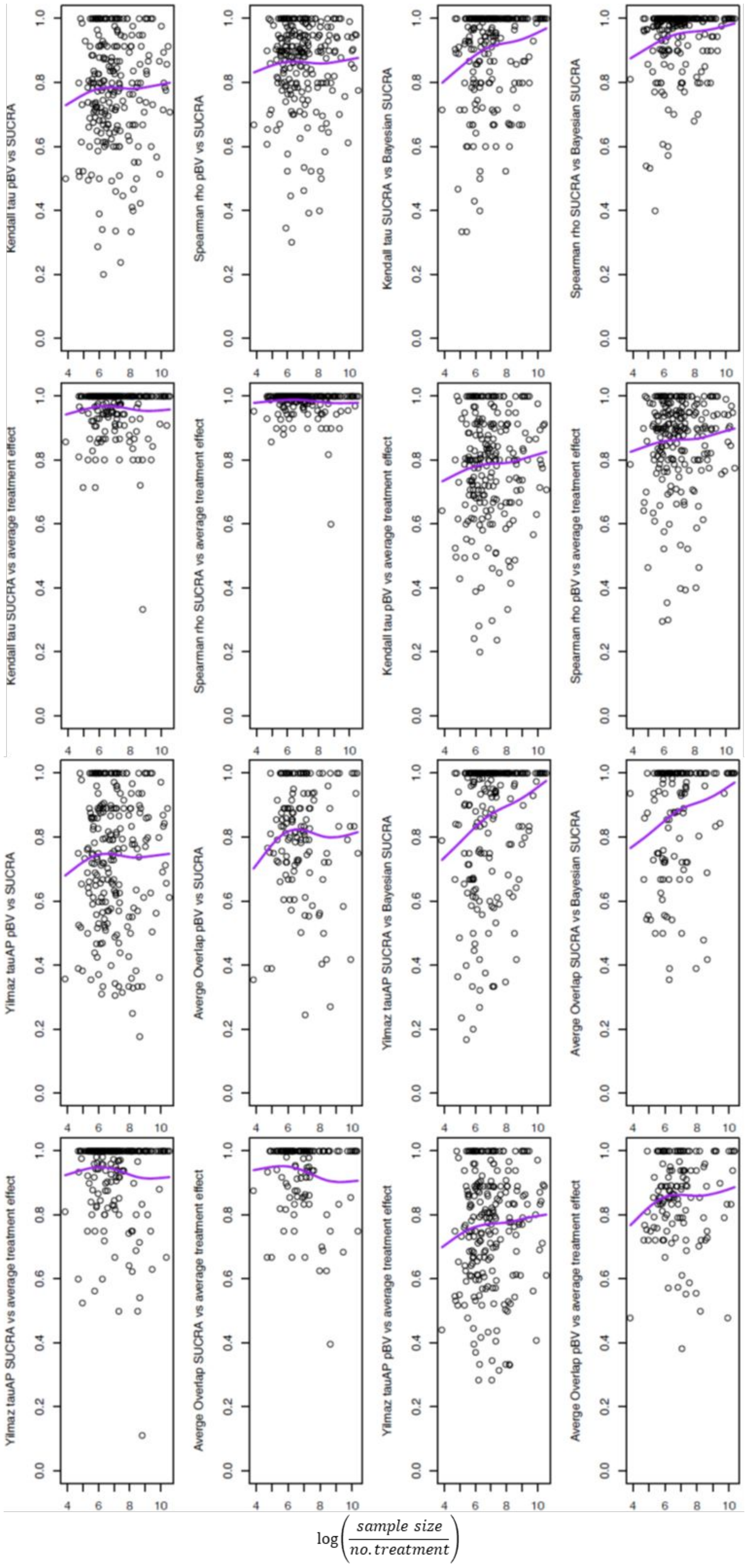

Figure S4: Scatter plots of the total sample size over the number of treatments in a network and the pairwise agreement between hierarchies from different ranking metrics.

Table S1: Pairwise agreement between treatment hierarchies from illustrative network example. Medians, 1st and 3rd quartiles are reported. p_{BV}: probability of producing the best value; $SUCRA_F$: surface under the cumulative ranking curve; relative treatment effect stands for the relative treatment effect against fictional treatment of average performance. The first three column from the left-hand side present the agreements between rankings obtained using the original data; the equivalent three columns on the right-hand side show the agreements between rankings obtained after reducing the standard error of the Conventional versus ARB treatment effect from 0.7 to a fictional value of 0.01.

	**Original data**			**Fictional data with increased precision for Conventional treatment versus ARB**		
	p_{BV} **vs** $SUCRA_F$	p_{BV} **vs relative treatment effect**	$SUCRA_F$ **vs relative treatment effect**	p_{BV} **vs** $SUCRA_F$	p_{BV} **vs relative treatment effect**	$SUCRA_F$ **vs relative treatment effect**
**Spearman ρ**	0.64	0.85	0.93	0.89	0.91	1
**Kendall τ**	0.54	0.69	0.87	0.78	0.82	0.99
**Yilmaz τ_{AP}**	0.39	0.44	0.87	0.76	0.81	0.96
**Average Overlap**	0.48	0.54	0.85	0.85	0.94	0.92

For peer review only

BMJ Open

Agreement between ranking metrics in network meta-analysis: an empirical study

Journal:	*BMJ Open*
Manuscript ID	bmjopen-2020-037744.R1
Article Type:	Original research
Date Submitted by the Author:	29-Jun-2020
Complete List of Authors:	Chiocchia, Virginia; University of Bern Institute of Social and Preventive Medicine<br>Nikolakopoulou, Adriani; University of Bern Institute of Social and Preventive Medicine<br>Papakonstantinou, Theodoros; University of Bern Institute of Social and Preventive Medicine<br>Egger, Matthias; University of Bern Institute of Social and Preventive Medicine<br>Salanti, Georgia; University of Bern Institute of Social and Preventive Medicine
**Primary Subject Heading**:	Epidemiology
Secondary Subject Heading:	Research methods
Keywords:	STATISTICS & RESEARCH METHODS, EPIDEMIOLOGY, PUBLIC HEALTH

SCHOLARONE™
Manuscripts

I, the Submitting Author has the right to grant and does grant on behalf of all authors of the Work (as defined in the below author licence), an exclusive licence and/or a non-exclusive licence for contributions from authors who are: i) UK Crown employees; ii) where BMJ has agreed a CC-BY licence shall apply, and/or iii) in accordance with the terms applicable for US Federal Government officers or employees acting as part of their official duties; on a worldwide, perpetual, irrevocable, royalty-free basis to BMJ Publishing Group Ltd ("BMJ") its licensees and where the relevant Journal is co-owned by BMJ to the co-owners of the Journal, to publish the Work in this journal and any other BMJ products and to exploit all rights, as set out in our licence.

The Submitting Author accepts and understands that any supply made under these terms is made by BMJ to the Submitting Author unless you are acting as an employee on behalf of your employer or a postgraduate student of an affiliated institution which is paying any applicable article publishing charge ("APC") for Open Access articles. Where the Submitting Author wishes to make the Work available on an Open Access basis (and intends to pay the relevant APC), the terms of reuse of such Open Access shall be governed by a Creative Commons licence – details of these licences and which Creative Commons licence will apply to this Work are set out in our licence referred to above.

Other than as permitted in any relevant BMJ Author's Self Archiving Policies, I confirm this Work has not been accepted for publication elsewhere, is not being considered for publication elsewhere and does not duplicate material already published. I confirm all authors consent to publication of this Work and authorise the granting of this licence.

For peer review only

Agreement between ranking metrics in network meta-analysis: an empirical study

Virginia Chiocchia[1], Adriani Nikolakopoulou[1], Theodoros Papakonstantinou[1], Matthias Egger[1], Georgia Salanti[1]

[1] Institute of Social and Preventive Medicine (ISPM), University of Bern, Bern, Switzerland

Correspondence to:

Virginia Chiocchia, Institute of Social and Preventive Medicine, University of Bern, Mittelstrasse 43, CH-3012 Bern, Switzerland.
Email: virginia.chiocchia@ispm.unibe.ch

Abstract 294 words, main text 3262 words, 3 tables, 3 figures, 26 references

For peer review only - http://bmjopen.bmj.com/site/about/guidelines.xhtml

Keywords: treatment hierarchy, multiple treatments, evidence synthesis, SUCRA, rank probabilities

For peer review only

For peer review only - http://bmjopen.bmj.com/site/about/guidelines.xhtml

ABSTRACT

Objective

To empirically explore the level of agreement of the treatment hierarchies from different ranking metrics in network meta-analysis (NMA) and to investigate how network characteristics influence the agreement.

Design

Empirical evaluation from re-analysis of network meta-analyses.

Data

232 networks of four or more interventions from randomised controlled trials, published between 1999 and 2015.

Methods

We calculated treatment hierarchies from several ranking metrics: relative treatment effects, probability of producing the best value (p_{BV}) and the surface under the cumulative ranking curve (SUCRA). We estimated the level of agreement between the treatment hierarchies using different measures: Kendall's τ and Spearman's ρ correlation; and the Yilmaz τ_{AP} and Average Overlap, to give more weight to the top of the rankings. Finally, we assessed how the amount of the information present in a network affects the agreement between treatment hierarchies, using the average variance, the relative range of variance, and the total sample size over the number of interventions of a network.

For peer review only - http://bmjopen.bmj.com/site/about/guidelines.xhtml

Results

Overall, the pairwise agreement was high for all treatment hierarchies obtained by the different ranking metrics. The highest agreement was observed between SUCRA and the relative treatment effect for both correlation and top-weighted measures whose medians were all equal to one. The agreement between rankings decreased for networks with less precise estimates and the hierarchies obtained from p_{BV} appeared to be the most sensitive to large differences in the variance estimates. However, such large differences were rare.

Conclusions

Different ranking metrics address different treatment hierarchy problems, however they produced similar rankings in the published networks. Researchers reporting NMA results can use the ranking metric they prefer, unless there are imprecise estimates or large imbalances in the variance estimates. In this case treatment hierarchies based on both probabilistic and non-probabilistic ranking metrics should be presented.

For peer review only

For peer review only - http://bmjopen.bmj.com/site/about/guidelines.xhtml

STRENGTH AND LIMITATIONS OF THIS STUDY

- To our knowledge, this is the first empirical study exploring the level of agreement of the treatment hierarchies from different ranking metrics in network meta-analysis (NMA).
- The study also explores how agreement is influenced by network characteristics.
- More than 200 published NMAs were re-analysed and three different ranking metrics calculated using both frequentist and Bayesian approaches.
- Other potential factors not investigated in this study could influence the agreement between hierarchies.

For peer review only

For peer review only - http://bmjopen.bmj.com/site/about/guidelines.xhtml

INTRODUCTION

Network meta-analysis (NMA) is being increasingly used by policy makers and clinicians to answer one of the key questions in medical decision-making: "what treatment works best for the given condition?" [1,2]. The relative treatment effects, estimated in NMA, can be used to produce ranking metrics: statistical quantities measuring the performance of an intervention on the studied outcomes, thus producing a treatment hierarchy from the most preferable to the least preferable option [3,4].

Despite the importance of treatment hierarchies in evidence-based decision making, various methodological issues related to the ranking metrics have been contested [5–7]. This ongoing methodological debate focuses on the uncertainty and bias in a single ranking metric. Hierarchies produced by different ranking metrics are not expected to agree because ranking metrics differ. For example, a *non-probabilistic ranking metric* such as the treatment effect against a common comparator considers only the mean effect (e.g. the point estimate of the odds-ratio) and ignores the uncertainty with which this is estimated. In contrast, the probability that a treatment achieves a specific rank (a *probabilistic ranking metric*) considers the entire estimated distribution of each treatment effect. However, it is important to understand why and how rankings based on different metrics differ.

There are network characteristics that are expected to influence the agreement of treatment hierarchies from different ranking metrics, such as the precision of the included studies and their distribution across treatment comparisons [4,8]. Larger imbalances in precision in the estimation of the treatment effects affects the agreement of the treatment hierarchies from probabilistic ranking metrics, but it is currently unknown whether in practice these imbalances occur and whether they should inform the choice between different ranking

For peer review only - http://bmjopen.bmj.com/site/about/guidelines.xhtml

metrics. To our knowledge, no empirical studies have explored the level of agreement of treatment hierarchies obtained from different ranking metrics, or examined the network characteristics likely to influence the level of agreement. Here, we empirically evaluated the level of agreement between ranking metrics and examined how the agreement is affected by network features. The article first describes the methods for the calculation of ranking metrics and of specific measures to assess the agreement and to explore factors that affects it, respectively. Then, a network featuring one of the explored factors is shown as an illustrative example to display differences in treatment hierarchies from different ranking metrics. Finally, we present the results from the empirical evaluation and discuss their implications for researchers undertaking network meta-analysis.

METHODS

Data

We re-analysed networks of randomised controlled trials from a database of articles published between 1999 and 2015, including at least 4 treatments; details about the search strategy and inclusion/exclusion criteria can be found in [9,10]. We selected networks reporting arm-level data for binary or continuous outcomes. The database is accessible in the *nmadb* R package [11].

Re-analysis and calculation of ranking metrics

All networks were re-analysed using the relative treatment effect that the original publication used: odds ratio (OR), risk ratio (RR), standardised mean difference (SMD) or mean difference (MD). We estimated relative effects between treatments using a frequentist random-effects

For peer review only - http://bmjopen.bmj.com/site/about/guidelines.xhtml

NMA model using the *netmeta* R package [12]. For the networks reporting ORs and SMDs we re-analysed them also using Bayesian models using self-programmed NMA routines in JAGS (https://github.com/esm-ispm-unibe-ch/NMAJags). To obtain probabilistic ranking metrics in a frequentist setting, we used parametric bootstrap by producing 1000 datasets from the estimated relative effects and their variance-covariance matrix. By averaging over the number of simulated relative effects we derived the *probability of treatment i to produce the best value*

$$p_{i,BV} := p_{i,1} = P(\mu_{ij} > 0 \ \forall j \in \mathbb{T})$$

where μ_{ij} is the estimated mean relative effect of treatment i against treatment j out of a set $\mathbb{T}$ of T competing treatments. We will refer to this as p_{BV}. This ranking metric indicates how likely a treatment is to produce the largest values for an outcome (or smallest value, if the outcome is harmful). We also calculated the surface under the cumulative ranking curve ($SUCRA^F$) [3]

$$SUCRA_i = \frac{\sum_{r=1}^{T-1} c_{i,r}}{T-1}$$

where $c_{i,r} = \sum_{v=1}^{r} p_{i,v}$ are the cumulative probabilities that treatment i will produce an outcome that is among the r best values (or that it outperforms $T - r$ treatments). SUCRA, unlike p_{BV}, also considers the probability of a treatment to produce unfavourable outcome values. Therefore, the treatment with the largest SUCRA value represents the one that outperforms the competing treatments in the network, meaning that overall it produces preferable outcomes compared to the others. We also obtained SUCRAs within a Bayesian framework ($SUCRA^B$).

To obtain the non-probabilistic ranking metric we fitted an NMA model and estimated related treatment effects. To obtain estimates for all treatments we reparametrize the NMA model

For peer review only - http://bmjopen.bmj.com/site/about/guidelines.xhtml

so that each treatment is compared to a fictional treatment of average performance [13,14]. The estimated relative effects against a fictional treatment *F* of average efficacy $\hat{\mu}_{iF}$ represent the ranking metric and the corresponding hierarchy is obtained simply by ordering the effects from the largest to the smallest (or in ascending order, if the outcome is harmful). The resulting hierarchy is identical to that obtained using relative effects from the conventional NMA model, irrespective of the reference treatment. In the rest of the manuscript, we will refer to this ranking metric simply as relative treatment effect.

Agreement between ranking metrics

To estimate the level of agreement between the treatment hierarchies obtained using the three chosen ranking methods we employed several correlation and similarity measures.

To assess the correlation between ranking metrics we used Kendall's τ [15] and the Spearman's ρ [16]. Both Kendall's τ and Spearman's ρ give the same weight to each item in the ranking. In the context of treatment ranking, the top of the ranking is more important than the bottom. We therefore also used a top-weighted variant of Kendall's τ, Yilmaz τ_{AP} [17], which is based on a probabilistic interpretation of the average precision measure used in information retrieval [18] (see online supplementary Appendix).

The measures described so far can only be considered for conjoint rankings, i.e. for lists where each item in one list is also present in the other list. Rankings are *non-conjoint* when a ranking is truncated to a certain *depth k* with such lists called *top-k rankings*. We calculated the Average Overlap [19,20], a top-weighted measure for top-k rankings that considers the cumulative intersection (or *overlap*) between the two lists and averages it over a specified depth (cut-off point) *k* (see online supplementary Appendix for details). We calculated the Average Overlap between pairs of rankings for networks with at least six treatments (139

For peer review only - http://bmjopen.bmj.com/site/about/guidelines.xhtml

networks) for a depth k equal to half the number of treatments in the network, $k = {}^{T}/_{2}$ (or $((T-1))/2$ if T is an odd number).

We calculated the four measures described above to assess the pairwise agreement between the three ranking metrics within the frequentist setting and summarised them for each pair of ranking metrics and each agreement measure using the median and the 1st and 3rd quartiles. The hierarchy according to $SUCRA^{B}$ was compared to that of its frequentist equivalent to check how often the two disagree.

Influence of network features on the rankings agreement

The main network characteristic considered was the amount of information in the network (reflected in the precision of the estimates). Therefore, for each network we calculated the following measures of information:

- the average variance, calculated as the mean of the variances of the estimated treatment effects $mean(SE^2)$, to show how much information is present in a network altogether;
- the relative range of variance, calculated as $\frac{\max SE^2 - \min SE^2}{\max SE^2}$, to describe differences in information about each intervention within the same networks;
- the total sample size of a network over the number of interventions.

These measures are presented in scatter plots against the agreement measurements for pairs of ranking metrics.

All the codes for the empirical evaluation are available at https://github.com/esm-ispm-unibe-ch/rankingagreement.

For peer review only - http://bmjopen.bmj.com/site/about/guidelines.xhtml

Patient and public involvement

Patients and the public were not involved in this study.

ILLUSTRATIVE EXAMPLE

To illustrate the impact of the amount of information on the treatment hierarchies from different ranking metrics, we used a network of nine antihypertensive treatments for primary prevention of cardiovascular disease that presents large differences in the precision of the estimates of overall mortality [21]. The network graph and forest plot of relative treatment effects of each treatment versus placebo are presented in **Figure 1**. The relative treatment effects reported are risk ratios (RR) estimated using a random effects NMA model.

Table 1 shows the treatment hierarchies obtained using the three ranking metrics described above. The highest overall agreement is between hierarchies from the $SUCRA^F$ and the relative treatment effect as shown by both correlation (Spearman's ρ = 0.93, Kendall's τ = 0.87) and top-weighted measures (Yilmaz's τ_{AP}= 0.87; Average Overlap = 0.85). The level of agreement decreases when $SUCRA^F$ and the relative treatment effect are compared with p_{BV} rankings (Spearman's ρ = 0.63 and ρ = 0.85 respectively). Agreement with p_{BV} especially decreases when considering top ranks only (Average Overlap is 0.48 for p_{BV} versus $SUCRA^F$ and 0.54 for p_{BV} versus relative treatment effect). All agreement measures are presented in online supplementary **Table S1**.

The reason for this disagreement is explained by the differences in precision in the estimated effects (**Figure 1**). These RRs versus placebo range from 0.82 (Diuretic/Beta-blocker versus placebo) to 0.98 (Beta-blocker versus placebo). All estimates are fairly precise except for the RR of conventional therapy versus placebo whose 95% confidence interval extends from 0.21

For peer review only - http://bmjopen.bmj.com/site/about/guidelines.xhtml

to 3.44. This uncertainty in the estimation is due to the fact that conventional therapy is compared only with Angiotensin Receptor Blockers (ARB) via a single study. This large difference in the precision of the estimation of the treatment effects mostly affects the p_{BV} ranking, which disagrees the most with both of the other rankings. Consequently, the Conventional therapy is in the first rank in the p_{BV} hierarchy (because of the large uncertainty) but only features in the third/fourth and sixth rank using the relative treatment effects and $SUCRA^F$ hierarchies, respectively.

To explore how the hierarchies for this network would change in case of increased precision, we reduced the standard error of the Conventional versus ARB treatment effect from the original 0.7 to a fictional value of 0.01 resulting in a confidence interval 0.77 to 0.96. The columns in the right-hand side of ***Table 2: Characteristics of the 232 NMAs included in the re-analysis.*** *display the three equivalent rankings after the standard error reduction. The conventional treatment has moved up in the hierarchy according to $SUCRA^F$ and moved down in the one based on p_{BV}, as expected. The treatment hierarchies obtained from the $SUCRA^F$ and the relative treatment effect are now identical (Conventional and ARB share the 3.5 rank because they have the same effect estimate) and the agreement with the p_{BV} rankings also improved (p_{BV} versus $SUCRA^F$ Spearman's ρ = 0.89, Average Overlap = 0.85; p_{BV} versus relative treatment effect Spearman's ρ = 0.91, Average Overlap = 0.94; online supplementary* ***Table S1****).*

RESULTS

A total of 232 networks were included in our dataset. Their characteristics are shown in **Table 2**. The majority of networks (133 NMAs, 57.3%) did not report any ranking metrics in the original publication. Among those which used a ranking metric to produce a treatment hierarchy, the probability of being the best was the most popular metric followed by the SUCRA with 35.8% and 6.9% of networks reporting them, respectively.

Table 3 presents the medians and quartiles for each similarity measures. All hierarchies showed a high level of pairwise agreement, although the hierarchies obtained from the $SUCRA^F$ and the relative treatment effect presented the highest values for both unweighted and with top-weighted measures (all measures' median equals 1). Only 4 networks (less than 2%) had a Spearman's correlation between $SUCRA^F$ and the relative treatment effect less

For peer review only - http://bmjopen.bmj.com/site/about/guidelines.xhtml

than 90% (not reported). The correlation becomes less between the p_{BV} rankings and those obtained from the other two ranking metrics with Spearman's ρ median decreasing to 0.9 and Kendall's τ decreasing to 0.8. The Spearman's correlation between these rankings was less than 90% in about 50% of the networks (in 116 and 111 networks for p_{BV} versus $SUCRA^F$ and p_{BV} versus relative effect, respectively; results not reported). The pairwise agreement between the p_{BV} rankings and the other rankings also decreased when considering only top ranks (p_{BV} versus $SUCRA^F$ Yilmaz's τ_{AP} = 0.77, Average Overlap = 0.83; p_{BV} versus relative treatment effect Yilmaz's τ_{AP} = 0.79, Average Overlap = 0.88).

The SUCRAs from frequentist and Bayesian settings ($SUCRA^F$ and $SUCRA^B$) were compared in 126 networks (82 networks using the Average Overlap measure) as these reported OR and SMD as original measures. The relevant rankings do not differ much as shown by the median values of the agreement measures all equal to 1 and their narrow interquartile ranges (Medians, 1st and 3rd quartiles are reported. p_{BV}: probability of producing the best value; $SUCRA_F$: surface under the cumulative ranking curve (calculated in frequentist setting); $SUCRA_B$: surface under the cumulative ranking curve (calculated in Bayesian setting); relative treatment effect stands for the relative treatment effect against fictional treatment of average performance.

). Nevertheless, a few networks showed a much lower agreement between the two SUCRAs. These networks provide posterior effect estimates for which the Normal approximation is not optimal, some of which due to rare outcomes. Such cases were however uncommon as in only 6% of the networks the Spearman's correlation between $SUCRA^F$ and $SUCRA^B$ was less than 90%. Plots for the Normal distributions from the frequentist setting and the posterior distributions of the log odds-ratios (LOR) for a network with a Spearman's ρ of 0.6 between the two SUCRAs is available in online supplementary **Figure S1** [22].

Figure 2 presents how Spearman's ρ and the Average Overlap vary with the average variance of the relative treatment effect estimates in a network (scatter plots for the Kendall's τ and the Yilmaz's τ_{AP} are available in online supplementary **Figure S2**). The treatment hierarchies agree more in networks with more precise estimates (left hand side of the plots).

For peer review only - http://bmjopen.bmj.com/site/about/guidelines.xhtml

The association between Spearman's ρ or Average Overlap and the relative range of variance in a network (here transformed to a double logarithm of the inverse values) are displayed in **Figure 3**. On the right-hand side of each plot we can find networks with smaller differences in the precision of the treatment effect estimates. Treatment hierarchies for these networks show a larger agreement than for those with larger differences in precision. The plots of the impact of the relative range of variance on all measures are available in online supplementary **Figure S3**.

The total sample size in a network over the number of interventions has a similar impact on the level of agreement between hierarchies. This confirms that the agreement between hierarchies increases for networks with a large total sample size compared to the number of treatments and, more generally, it increases with the amount of information present in a network (online supplementary **Figure S4**).

DISCUSSION

Our empirical evaluation showed that in practice the level of agreement between treatment hierarchies is overall high for all ranking metrics used. The agreement between treatment hierarchies from $SUCRA$ and relative treatment effect was very often perfect. The agreement between the rankings from $SUCRA$ or relative treatment effect and the ranking from p_{BV} was good but decreased when the top-ranked interventions are of interest. The agreement is higher for networks with precise estimates and small imbalances in precision.

Simulation studies [6,23] using theoretical examples have shown the importance of accounting for the precision in the estimation of the treatment effects when a hierarchy is to

For peer review only - http://bmjopen.bmj.com/site/about/guidelines.xhtml

For peer review only

be obtained. However, we show that cases of extreme imbalance in the precision of the treatment effects are rather uncommon.

Several factors can be responsible for imprecision in the estimation of the relative treatment effects in a network:

- large sampling error, determined by a small sample size, small number of events or a large standard deviation;
- poor connectivity of the network, when only a few links and few closed loops of evidence connect the treatments;
- residual inconsistency;
- heterogeneity in the relative treatment effects.

Random-effects models tend to provide relative treatment effects with similar precision as heterogeneity increases. In contrast, in the absence of heterogeneity when fixed-effects models are used, the precision of the effects can vary a lot according to the amount of data available for each intervention. In the latter case, the ranking metrics are likely to disagree. Also, the role of precision in ranking disagreement is more pronounced in cases where the interventions have similar effects.

Our results also confirm that a treatment hierarchy can differ when the uncertainty in the estimation is incorporated into the ranking metric (by using, for example, a probabilistic metric rather than ranking the point estimate of the mean treatment effect) [8,24] and that rankings from the p_{BV} seem to be the most sensitive to differences in precision in the estimation of treatment effects. We showed graphically that the agreement is less in networks with more uncertainty and with larger imbalances in the variance estimates. However, we also found that such large imbalances do not occur frequently in real data and in the majority of cases the different treatment hierarchies have a relatively high agreement.

For peer review only - http://bmjopen.bmj.com/site/about/guidelines.xhtml

We acknowledge that there could be other factors influencing the agreement between hierarchies that we did not explore, such as the chosen effect measures [25]. However, we think it is unlikely that such features play a big role in ranking agreement unless assumptions are violated or data in the network is sparse [26]. Adjustment via network meta-regression (for example, for risk of bias or small-study effects) might impact on the ranking of treatments not only by changing the point estimate but also by altering the total precision and the imbalance in the precision of the estimated treatment effects. We did not investigate the agreement between treatment hierarchies obtained from such adjusted analyses. We also did not explore non-methodological characteristics for networks with larger disagreement but we believe these characteristics are a proxy for the amount of information in a network, which is the main factor affecting the agreement between ranking metrics. For example, in some specific fields there are few or small randomised trials (e.g. surgery) and, as a consequence, the resulting networks will have less information. Also, smaller (hence more imprecise) networks might be published more often in journal with lower impact factor and get less citations than large and precise networks.

To our knowledge, this is the first empirical study assessing the level of agreement between treatment hierarchies from ranking metrics in NMA and it provides further insights into the properties of the different methods. In this context, it is important to stress that neither the objective nor the findings of this empirical evaluation imply that a hierarchy for a particular metric works better or is more accurate than one obtained from another ranking metric. The reason why this sort of comparison cannot be made is that each ranking metric address a specific treatment hierarchy problem. For example, the $SUCRA$ ranking addresses the issue of which treatment outperforms most of the competing interventions, while the ranking

For peer review only - http://bmjopen.bmj.com/site/about/guidelines.xhtml

based on the relative treatment effect gives an answer to the problem of which treatment is associated with the largest average effect for the outcome considered.

Our study shows that, despite theoretical differences between ranking metrics and some extreme examples, they produce very similar treatment hierarchies in published networks. In networks with large amount of data for each treatment, hierarchies based on SUCRA or the relative treatment effect will almost always agree. Large imbalances in the precision of the treatment effect estimates do not occur often enough to motivate a choice between the different ranking metrics. Therefore, our advice to researchers presenting results from NMA is the following: *if the NMA estimated effects are precise,* to use the ranking metric they prefer; *if at least one NMA estimated effect is imprecise,* to refrain from making bold statements about treatment hierarchy and present hierarchies from both probabilistic (e.g. SUCRA or rank probabilities) and non-probabilistic metrics (e.g. relative treatments effects).

Author contributions

VC designed the study, analysed the data, interpreted the results of the empirical evaluation, and drafted the manuscript. GS designed the study, interpreted the results of the empirical evaluation and revised the manuscript. AN provided input into the study design and the data analysis, interpreted the results of the empirical evaluation and revised the manuscript. TP developed and manages the database where networks' data was accessed, provided input into the data analysis and revised the manuscript. ME provided input into the study design and revised the manuscript. All the authors approved the final version of the submitted manuscript.

For peer review only - http://bmjopen.bmj.com/site/about/guidelines.xhtml

Funding

This work was supported by the Swiss National Science Foundation grant/award number 179158.

Competing Interests

All authors have completed the ICMJE uniform disclosure form and declare: all authors had financial support from the Swiss National Science Foundation for the submitted work; no financial relationships with any organizations that might have an interest in the submitted work in the previous three years; no other relationships or activities that could appear to have influenced the submitted work.

Data sharing statement

The data for the network meta-analyses included in this study are available in the database accessible using the *nmadb* R package.

Statement of Ethics Approval

Ethical approval was not required as human participants were not involved in this study.

For peer review only

References

1 Efthimiou O, Debray TPA, van Valkenhoef G, *et al.* GetReal in network meta-analysis: a review of the methodology: reviewNMA. *Res Synth Methods* 2016;**7**:236–63. doi:10.1002/jrsm.1195

2 Del Fiol G, Workman TE, Gorman PN. Clinical questions raised by clinicians at the point of care: a systematic review. *JAMA Intern Med* 2014;**174**:710–8. doi:10.1001/jamainternmed.2014.368

3 Salanti G, Ades AE, Ioannidis JPA. Graphical methods and numerical summaries for presenting results from multiple-treatment meta-analysis: an overview and tutorial. *J Clin Epidemiol* 2011;**64**:163–71. doi:10.1016/j.jclinepi.2010.03.016

4 Rücker G, Schwarzer G. Ranking treatments in frequentist network meta-analysis works without resampling methods. *BMC Med Res Methodol* 2015;**15**:58. doi:10.1186/s12874-015-0060-8

5 Trinquart L, Attiche N, Bafeta A, *et al.* Uncertainty in Treatment Rankings: Reanalysis of Network Meta-analyses of Randomized Trials. *Ann Intern Med* 2016;**164**:666–73. doi:10.7326/M15-2521

6 Kibret T, Richer D, Beyene J. Bias in identification of the best treatment in a Bayesian network meta-analysis for binary outcome: a simulation study. *Clin Epidemiol* 2014;**6**:451–60. doi:10.2147/CLEP.S69660

7 Veroniki AA, Straus SE, Rücker G, *et al.* Is providing uncertainty intervals in treatment ranking helpful in a network meta-analysis? *J Clin Epidemiol* 2018;**100**:122–9. doi:10.1016/j.jclinepi.2018.02.009

8 Jansen JP, Trikalinos T, Cappelleri JC, *et al.* Indirect Treatment Comparison/Network Meta-Analysis Study Questionnaire to Assess Relevance and Credibility to Inform Health Care Decision Making: An ISPOR-AMCP-NPC Good Practice Task Force Report. *Value Health* 2014;**17**:157–73. doi:10.1016/j.jval.2014.01.004

9 Petropoulou M, Nikolakopoulou A, Veroniki A-A, *et al.* Bibliographic study showed improving statistical methodology of network meta-analyses published between 1999 and 2015. *J Clin Epidemiol* 2017;**82**:20–8. doi:10.1016/j.jclinepi.2016.11.002

10 Nikolakopoulou A, Chaimani A, Veroniki AA, *et al.* Characteristics of networks of interventions: a description of a database of 186 published networks. *PloS One* 2014;**9**:e86754. doi:10.1371/journal.pone.0086754

11 Papakonstantinou T. *nmadb: Network Meta-Analysis Database API.* 2019. https://CRAN.R-project.org/package=nmadb

12 Rücker G, Krahn U, König J, *et al. netmeta: Network Meta-Analysis using Frequentist Methods.* 2019. https://github.com/guido-s/netmeta http://meta-analysis-with-r.org.

13 Hosmer DW, Lemeshow S. *Applied Logistic Regression: Hosmer/Applied Logistic Regression*. Hoboken, NJ, USA: : John Wiley & Sons, Inc. 2000. doi:10.1002/0471722146

14 Nikolakopoulou A, Mavridis D, Chiocchia V, *et al.* PreTA: A network meta-analysis ranking metric measuring the probability of being preferable than the average treatment. *Res Synth Methods* (submitted).

15 Kendall MG. THE TREATMENT OF TIES IN RANKING PROBLEMS. *Biometrika* 1945;**33**:239–51. doi:10.1093/biomet/33.3.239

16 Spearman C. The Proof and Measurement of Association between Two Things. *Am J Psychol* 1904;**15**:72. doi:10.2307/1412159

For peer review only - http://bmjopen.bmj.com/site/about/guidelines.xhtml

17 Yilmaz E, Aslam JA, Robertson S. A new rank correlation coefficient for information retrieval. In: *Proceedings of the 31st annual international ACM SIGIR conference on Research and development in information retrieval - SIGIR '08*. Singapore, Singapore: : ACM Press 2008. 587. doi:10.1145/1390334.1390435

18 Yilmaz E, Aslam JA. Estimating Average Precision with Incomplete and Imperfect Judgments. In: *Proceedings of the 15th ACM International Conference on Information and Knowledge Management*. New York, NY, USA: : ACM 2006. 102–111. doi:10.1145/1183614.1183633

19 Fagin R, Kumar R, Sivakumar D. Comparing Top K Lists. In: *Proceedings of the Fourteenth Annual ACM-SIAM Symposium on Discrete Algorithms*. Philadelphia, PA, USA: : Society for Industrial and Applied Mathematics 2003. 28–36.http://dl.acm.org/citation.cfm?id=644108.644113 (accessed 15 May 2019).

20 Wu S, Crestani F. Methods for Ranking Information Retrieval Systems Without Relevance Judgments. In: *Proceedings of the 2003 ACM Symposium on Applied Computing*. New York, NY, USA: : ACM 2003. 811–816. doi:10.1145/952532.952693

21 Fretheim A, Odgaard-Jensen J, Brørs O, *et al.* Comparative effectiveness of antihypertensive medication for primary prevention of cardiovascular disease: systematic review and multiple treatments meta-analysis. *BMC Med* 2012;**10**:33. doi:10.1186/1741-7015-10-33

22 Greco T, Calabrò MG, Covello RD, *et al.* A Bayesian network meta-analysis on the effect of inodilatory agents on mortality. *Br J Anaesth* 2015;**114**:746–56. doi:10.1093/bja/aeu446

23 Davies AL, Galla T. Degree irregularity and rank probability bias in network meta-analysis. *ArXiv200307662 Cond-Mat Stat* Published Online First: 17 March 2020.http://arxiv.org/abs/2003.07662 (accessed 24 Jun 2020).

24 Chaimani A, Vasiliadis HS, Pandis N, *et al.* Effects of study precision and risk of bias in networks of interventions: a network meta-epidemiological study. *Int J Epidemiol* 2013;**42**:1120–31. doi:10.1093/ije/dyt074

25 Norton EC, Miller MM, Wang JJ, *et al.* Rank Reversal in Indirect Comparisons. *Value Health* 2012;**15**:1137–40. doi:10.1016/j.jval.2012.06.001

26 van Valkenhoef G, Ades AE. Evidence Synthesis Assumes Additivity on the Scale of Measurement: Response to "Rank Reversal in Indirect Comparisons" by Norton et al. *Value Health* 2013;**16**:449–51. doi:10.1016/j.jval.2012.11.012

For peer review only

For peer review only - http://bmjopen.bmj.com/site/about/guidelines.xhtml

Table 1: Example of treatment hierarchies from different ranking metrics for a network of nine antihypertensive treatment for primary prevention of cardiovascular disease.

Treatment	Original data			Fictional data with increased precision for Conventional treatment versus ARB		
	p_{BV} ranks	$SUCRA_F$ ranks	Relative treatment effect ranks	p_{BV} ranks	$SUCRA_F$ ranks	Relative treatment effect ranks
**Conventional**	1	6	3.5	3	4	3.5
**Diuretic/Beta-blocker**	2	1	1	1	1	1
**ARB**	3	3	3.5	4.5	3	3.5
**CCB**	4	2	2	2	2	2
**Alpha-blocker**	5	7	7	4.5	7	7
**ACE-inhibitor**	6	4	5	6.5	5	5
**Diuretic**	7	5	6	6.5	6	6
**Placebo**	8.5	9	9	8.5	9	9
**Beta-Blocker**	8.5	8	8	8.5	8	8

ACE=Angiotensin Converting Enzyme; CCB=Calcium Channel Blockers; ARB=Angiotensin Receptor Blockers. p_{BV}: probability of producing the best value; $SUCRA_F$: surface under the cumulative ranking curve (calculated in frequentist setting); relative treatment effect stands for the relative treatment effect against fictional treatment of average performance. The first three rankings from the left-hand side are obtained using the original data; the equivalent three rankings on the right-hand side are produced by reducing the standard error of the Conventional versus ARB treatment effect from 0.7 to a fictional value of 0.01.

Table 2: Characteristics of the 232 NMAs included in the re-analysis.

Characteristics of networks	Median	IQR
Median number of treatments compared	6	(5, 9)
Median number of studies included	19	(12, 34)
Median total sample size	6100	(2514, 17264)
	Number of NMAs	%
Beneficial outcome	97	41.8%
Dichotomous outcome	185	79.7%
Continuous outcome	47	20.3%
Published before 2010	42	18.1%
Ranking metric used in original publication (non-exclusive):		
Probability of producing the best value	83	35.8%
Rankograms	7	3%
Median or mean rank	3	1.3%
SUCRA	16	6.9%
Other	2	0.9%
None	133	57.3%

For peer review only - http://bmjopen.bmj.com/site/about/guidelines.xhtml

**Published in general medicine journals†**	125	53.9%
**Published in health services research journals‡**	3	1.3%
**Published in specialty journals**	104	44.8%

IQR: interquartile range; NMA: network meta-analysis; SUCRA: surface under the cumulative ranking curve.
† Includes the categories Medicine, General & Internal, Pharmacology & Pharmacy, Research & Experimental, Primary Health Care.
‡ Includes the categories Health Care Sciences & Services, Health Policy & Services.

Table 3: Pairwise agreement between treatment hierarchies obtained from the different ranking metrics measured by Spearman ρ, Kendall τ, Yilmaz τ_{AP} and Average Overlap.

	p_{BV} vs $SUCRA_F$	$SUCRA_F$ vs relative treatment effect	p_{BV} vs relative treatment effect	$SUCRA_F$ vs $SUCRA_B$
**Spearman ρ**	0.9 (0.8, 0.96)	1 (0.99, 1)	0.9 (0.8, 0.97)	1 (0.98, 1)
**Kendall τ**	0.8 (0.67, 0.91)	1 (0.95, 1)	0.8 (0.69, 0.91)	1 (0.93, 1)
**Yilmaz τ_{AP}**	0.78 (0.6, 0.9)	1 (0.93, 1)	0.79 (0.65, 0.9)	1 (0.93, 1)
**Average Overlap**	0.85 (0.72, 0.96)	1 (0.91, 1)	0.88 (0.79, 1)	1 (0.94, 1)

Medians, 1st and 3rd quartiles are reported. p_{BV}: probability of producing the best value; $SUCRA_F$: surface under the cumulative ranking curve (calculated in frequentist setting); $SUCRA_B$: surface under the cumulative ranking curve (calculated in Bayesian setting); relative treatment effect stands for the relative treatment effect against fictional treatment of average performance.

Figure 1: (left panel) Network graph of network of nine antihypertensive treatments for primary prevention of cardiovascular disease. *Line width is proportional to inverse standard error of random effects model comparing two treatments.* ***(right panel) Forest plots of relative treatment effects of overall mortality for each treatment versus placebo.*** *RR: risk ratio; ACE=Angiotensin Converting Enzyme; CCB=Calcium Channel Blockers; ARB=Angiotensin Receptor Blockers; SE=standard error.*

Figure 2: Scatter plots of the average variance in a network and the pairwise agreement between hierarchies from different ranking metrics. *The average variance is calculated as the mean of the variances of the estimated treatment effects and describes the average information present in a network. More imprecise network are on the right-hand side of the plots. Spearman ρ (top row) and Average Overlap (bottom row) values for the pairwise agreement between p_{BV} and SUCRA (first column), SUCRA and relative treatment effect (second column), p_{BV} and relative treatment effect (third column). Purple line: cubic smoothing spline with five degrees of freedom.*

Figure 3: Scatter plots of the relative range of variance in a network and the pairwise agreement between hierarchies from different ranking metrics. *The relative range of variance, calculated as $\frac{max\,SE^2 - min\,SE^2}{max\,SE^2}$, indicates how much the information differs between interventions in the same networks. Networks with larger differences in variance are on the left-hand side of the plots. Spearman ρ (top row) and Average Overlap (bottom row) values for the pairwise agreement between p_{BV} and SUCRA (first column), SUCRA and relative treatment effect (second column), p_{BV} and relative treatment effect (third column). Purple line: cubic smoothing spline with five degrees of freedom.*

For peer review only - http://bmjopen.bmj.com/site/about/guidelines.xhtml

For peer review only

For peer review only - http://bmjopen.bmj.com/site/about/guidelines.xhtml

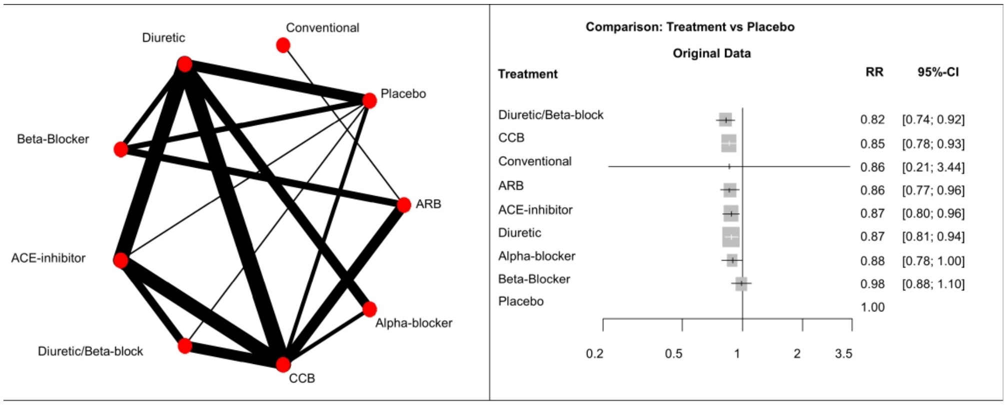

Figure 1: (left panel) Network graph of network of nine antihypertensive treatments for primary prevention of cardiovascular disease. Line width is proportional to inverse standard error of random effects model comparing two treatments. **(right panel) Forest plots of relative treatment effects of overall mortality for each treatment versus placebo.** RR: risk ratio; ACE=Angiotensin Converting Enzyme; CCB=Calcium Channel Blockers; ARB=Angiotensin Receptor Blockers; SE=standard error.

338x134mm (300 x 300 DPI)

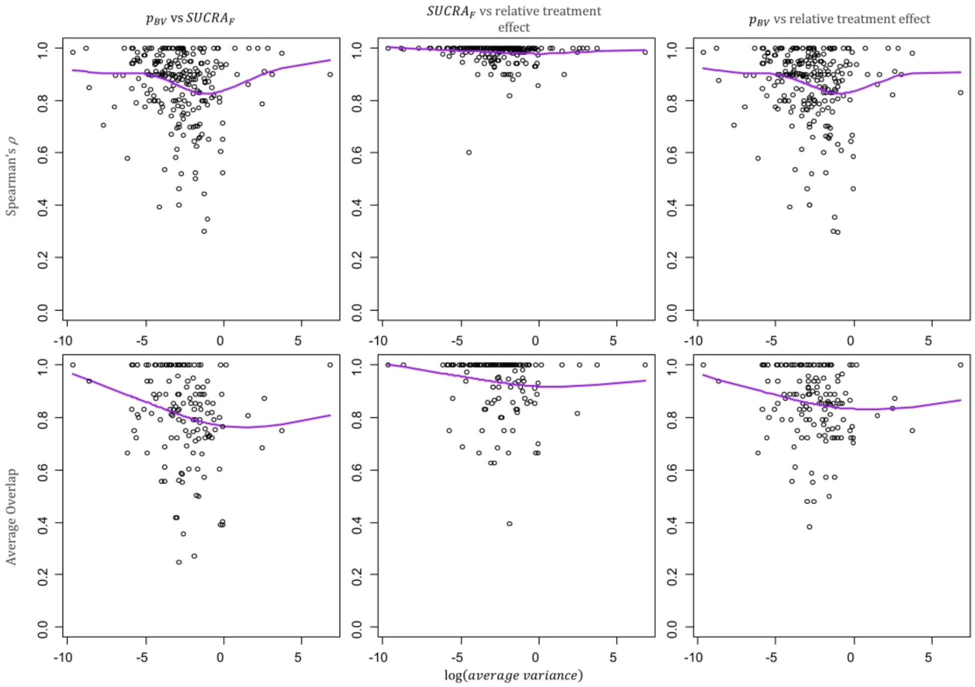

Figure 2: Scatter plots of the average variance in a network and the pairwise agreement between hierarchies from different ranking metrics. The average variance is calculated as the mean of the variances of the estimated treatment effects and describes the average information present in a network. More imprecise network are on the right-hand side of the plots. Spearman ρ (top row) and Average Overlap (bottom row) values for the pairwise agreement between p_{BV} and SUCRA (first column), SUCRA and relative treatment effect (second column), p_{BV} and relative treatment effect (third column). Purple line: cubic smoothing spline with five degrees of freedom.

270x190mm (300 x 300 DPI)

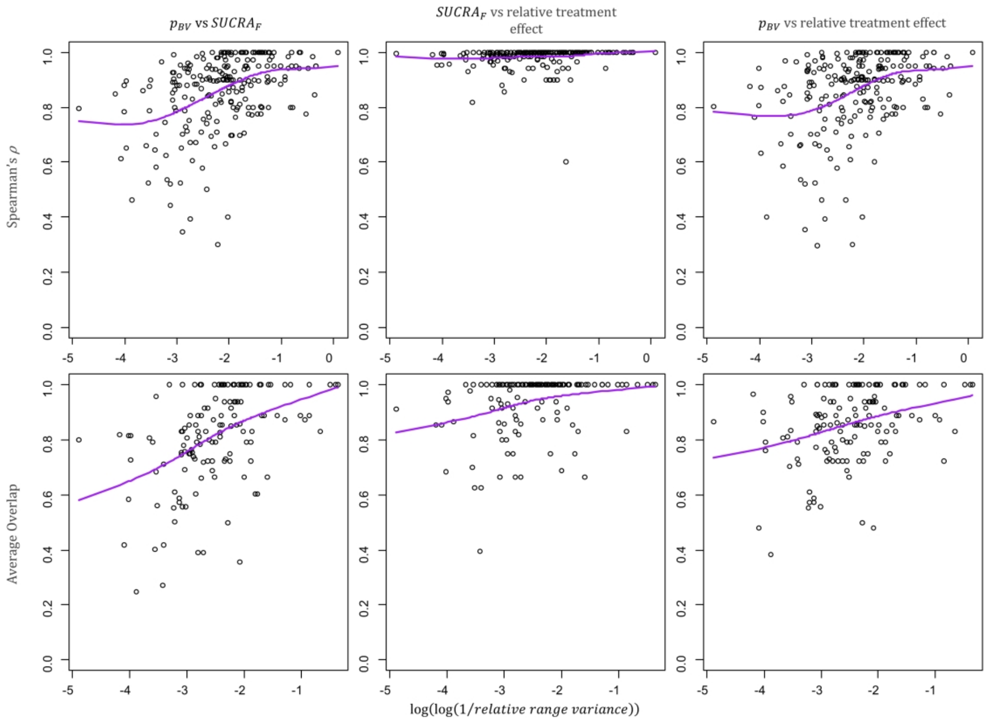

Figure 3: Scatter plots of the relative range of variance in a network and the pairwise agreement between hierarchies from different ranking metrics. The relative range of variance, calculated as $(\max(SE^2) - \min(SE^2)) / \max(SE^2)$, indicates how much the information differs between interventions in the same networks. Networks with larger differences in variance are on the left-hand side of the plots. Spearman ρ (top row) and Average Overlap (bottom row) values for the pairwise agreement between p_{BV} and SUCRA (first column), SUCRA and relative treatment effect (second column), p_{BV} and relative treatment effect (third column). Purple line: cubic smoothing spline with five degrees of freedom.

261x190mm (300 x 300 DPI)

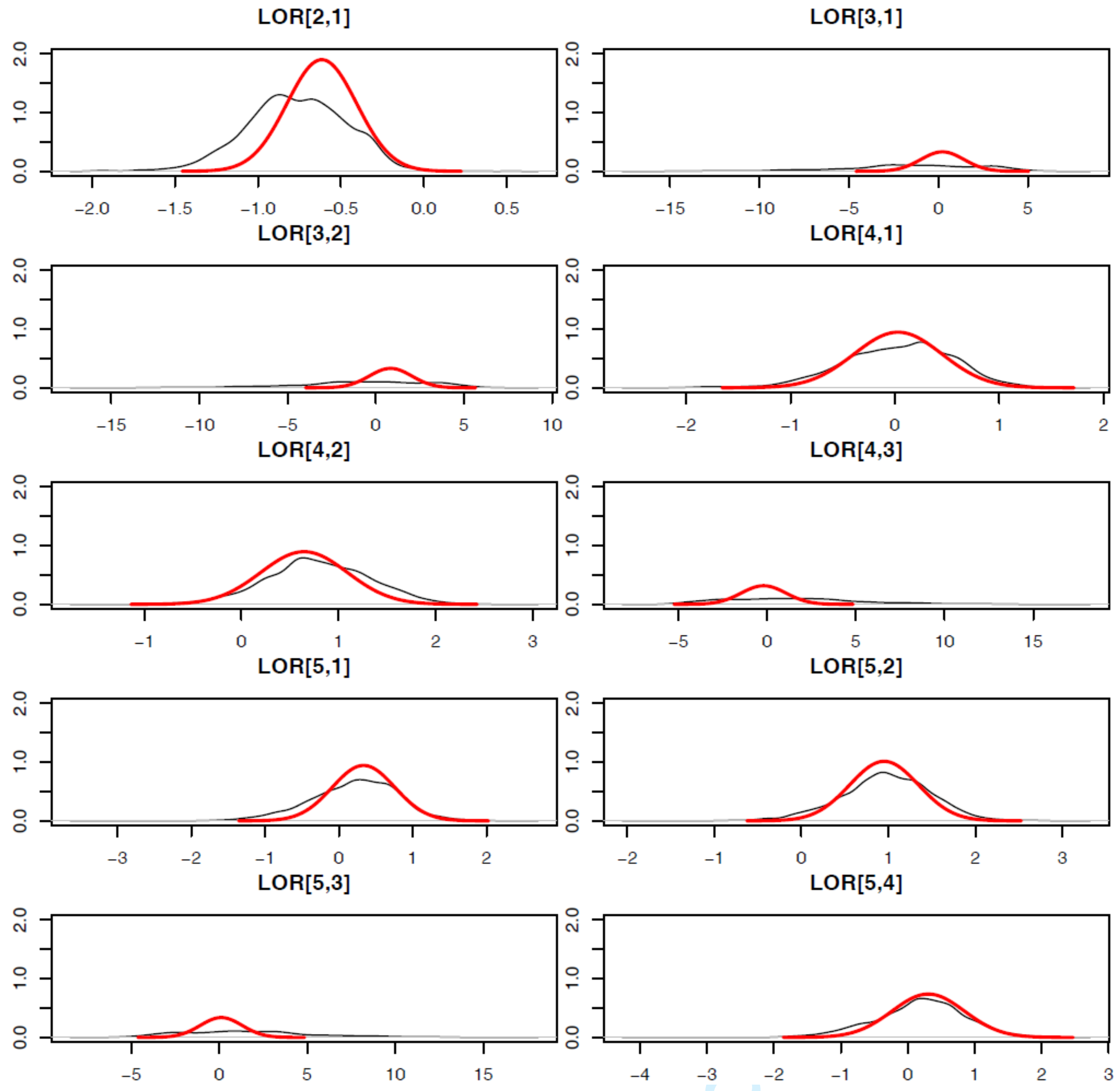

Figure S1: Normal distributions of relative treatment effect estimates from frequentist setting (red line) superimposed on posterior distributions of the relative treatment effects from Bayesian model (black line) for a network with Spearman's ρ of 0.6 between $SUCRA_F$ and $SUCRA_B$. The network meta-analysis used analysed the effects of four inodilators and placebo on survival in adult cardiac surgery patients (Greco et al., *Br J Anaesth* 2015). LOR = log odds ratios; number in square brackets represents the different interventions in the network.

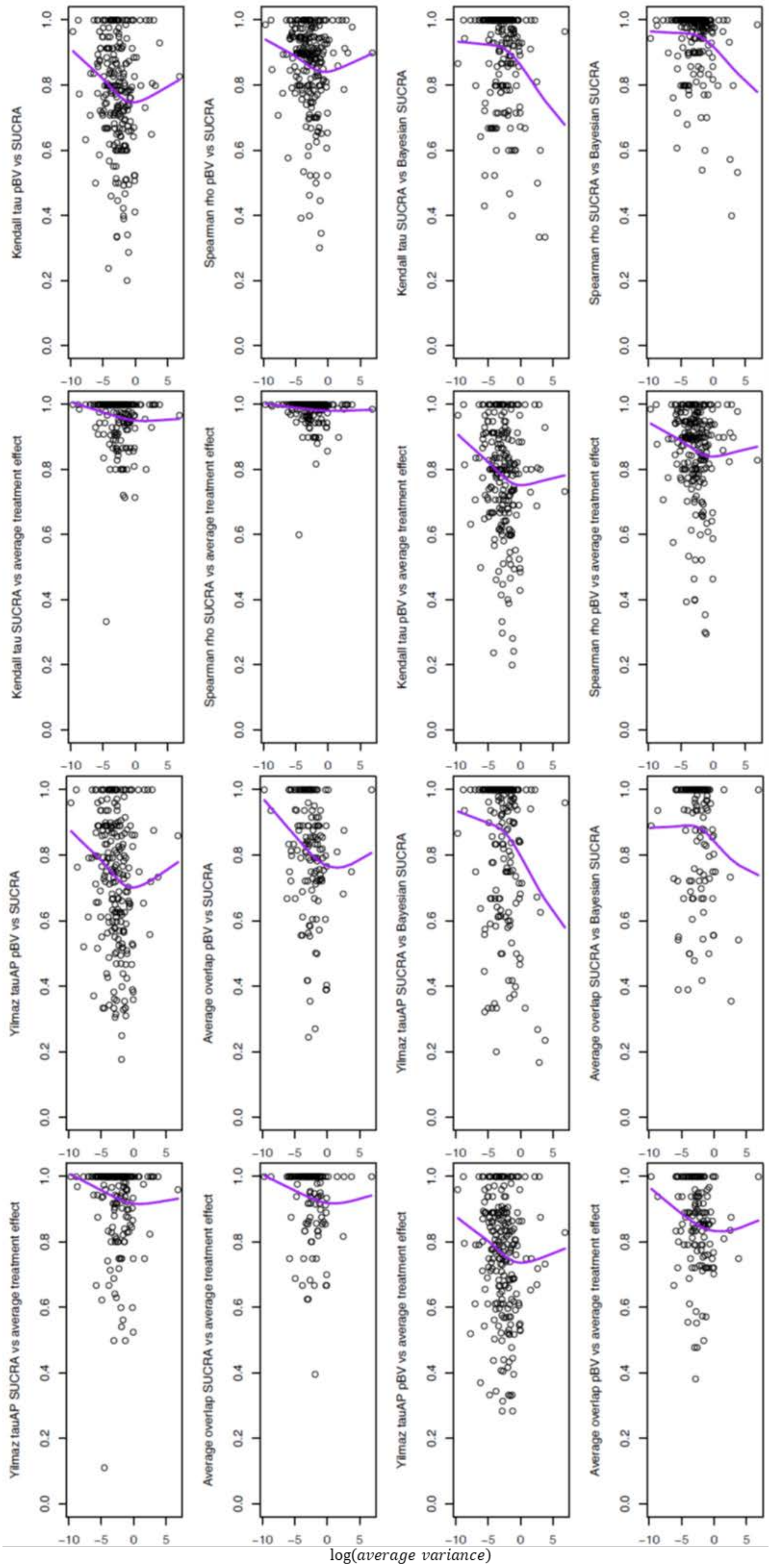

Figure S2: Scatter plots of the average variance in a network and the pairwise agreement between hierarchies from different ranking metrics.

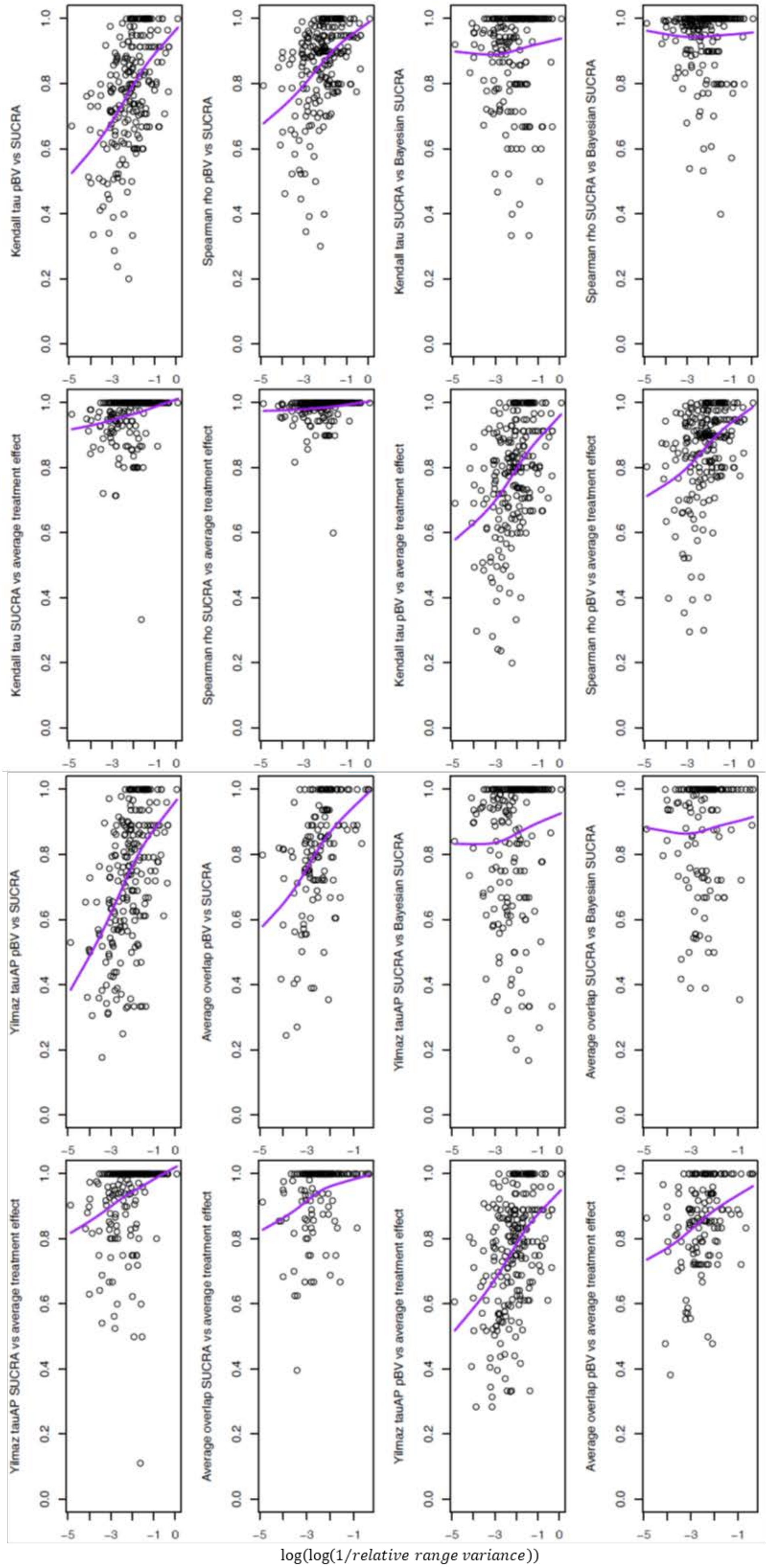

Figure S3: Scatter plots of the relative range of variance in a network and the pairwise agreement between hierarchies from different ranking metrics.

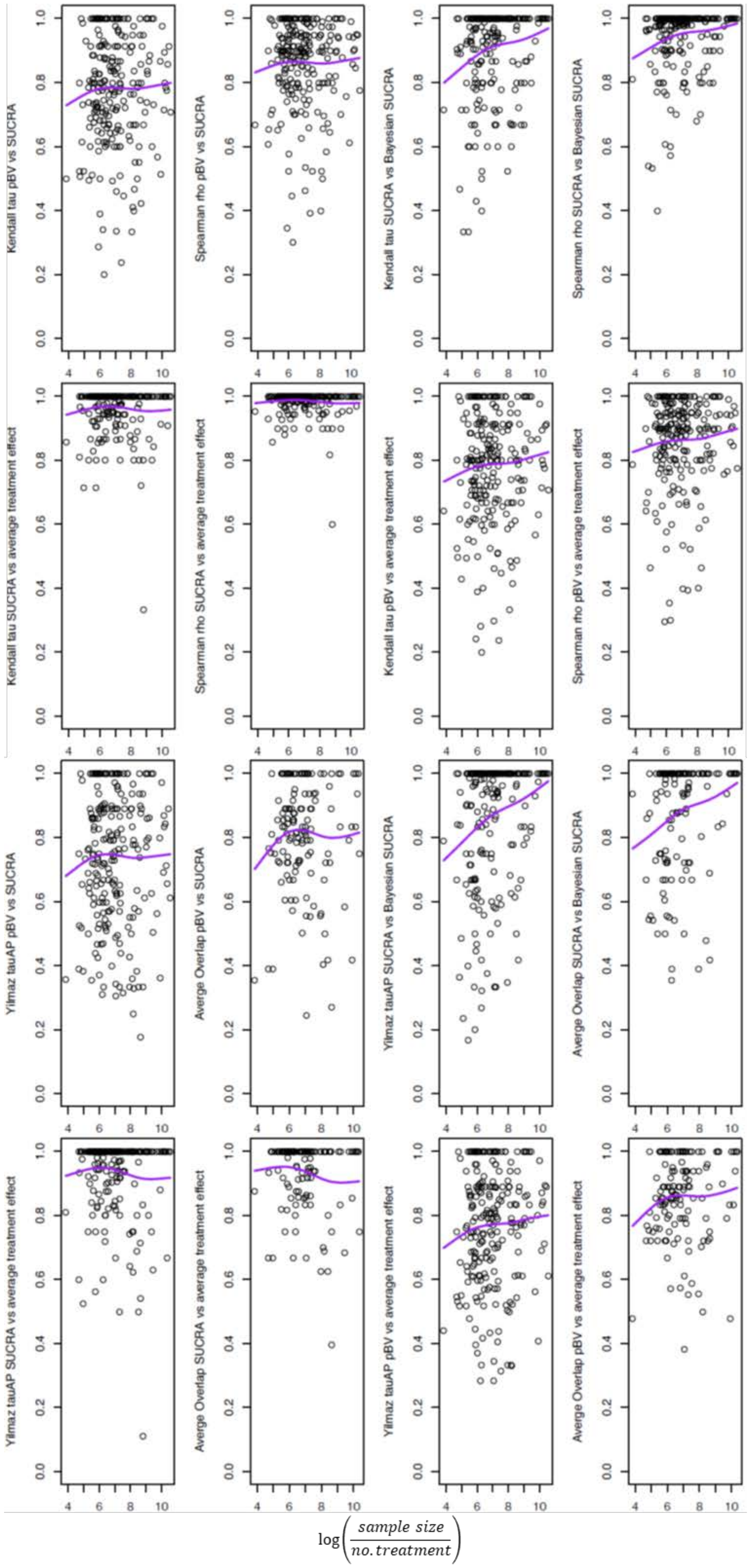

Figure S4: Scatter plots of the total sample size over the number of treatments in a network and the pairwise agreement between hierarchies from different ranking metrics.

Table S1: Pairwise agreement between treatment hierarchies from illustrative network example. *Medians, 1st and 3rd quartiles are reported. p_{BV}: probability of producing the best value; $SUCRA_F$: surface under the cumulative ranking curve; relative treatment effect stands for the relative treatment effect against fictional treatment of average performance. The first three column from the left-hand side present the agreements between rankings obtained using the original data; the equivalent three columns on the right-hand side show the agreements between rankings obtained after reducing the standard error of the Conventional versus ARB treatment effect from 0.7 to a fictional value of 0.01.*

	Original data			Fictional data with increased precision for Conventional treatment versus ARB		
	p_{BV} vs $SUCRA_F$	p_{BV} vs relative treatment effect	$SUCRA_F$ vs relative treatment effect	p_{BV} vs $SUCRA_F$	p_{BV} vs relative treatment effect	$SUCRA_F$ vs relative treatment effect
**Spearman ρ**	0.64	0.85	0.93	0.89	0.91	1
**Kendall τ**	0.54	0.69	0.87	0.78	0.82	0.99
**Yilmaz τ_{AP}**	0.39	0.44	0.87	0.76	0.81	0.96
**Average Overlap**	0.48	0.54	0.85	0.85	0.94	0.92

For peer review only

APPENDIX – YILMAZ'S τ_{AP} AND AVERAGE OVERLAP

The Yilmaz's τ_{AP} calculates the difference between the probability of observing concordance and the probability of observing discordance between two rankings X and Y, penalising more the discordance between top ranks. It can be computed as

$$\tau_{AP}(X,Y) = \frac{2}{N-1}\sum_{i=2}^{N}\sum_{j<i}\frac{C_{ij}}{i-1} - 1$$

where cij is 1 in case the items i and j are concordant and 0 otherwise; N is the total number of items in the ranking.

As Yilmaz's τ_{AP} is not symmetric, the authors proposed an alternative measure that takes the average between the two τ_{AP}, with the second being the one calculated after swapping the two rankings

$$symm\,\tau_{AP}(X,Y) = \left(\tau_{AP}(X|Y) + \tau_{AP}(Y|X)\right)/2$$

As with the original Kendall's τ, also the Yilmaz's τ_{AP} formula above does not handle ties. Similarly, two formulations to account for this have been proposed[i] and we selected the one that considers correlation as a measure of agreement because more relevant for our purpose. In our chosen version of the Yilmaz's τ_{AP}, the $\tau_{AP,b}$, neither of the two rankings is considered "true and objective" and ties can be present in either or both of them. The formula appears as follows

$$\tau_{AP,b} = \left(\tau_{AP,ties}(X|Y) + \tau_{AP,ties}(Y|X)\right)/2 \qquad \tau_{AP,ties} = \frac{2}{n-t_1}\sum_{i=t_1+1}^{n}\sum_{i<p_i}\frac{C_{ij}}{p_i-1} - 1$$

where t_1 is the number of items tied in position i=1 and pi is the rank of the first item in i's group.

The Average Overlap is a top-weighted measure for top-k rankings that considers the intersection (or overlap) between the two lists, $|X \cap Y|/k$. It calculates the cumulative overlap at increasing depths d, d ∊ {1…k} and average it over the depth (cut-off point) k.

$$AO(X,Y,k) = \frac{1}{k}\sum_{d=1}^{k} A_d \qquad \text{where } A_d = |X \cap Y|/d$$

Unlike the previous measures, the average overlap takes values between 0 and 1.

[i] Julián Urbano and Mónica Marrero, 'The Treatment of Ties in AP Correlation', in *Proceedings of the ACM SIGIR International Conference on Theory of Information Retrieval - ICTIR '17* (the ACM SIGIR International Conference, Amsterdam, The Netherlands: ACM Press, 2017), 321–24, https://doi.org/10.1145/3121050.3121106.

For peer review only
